# Molecular mechanism of resistance to lonafarnib conferred by mutations in the cysteine-rich region of respiratory syncytial virus fusion glycoprotein and discovery of a lonafarnib-derived antiviral PROTAC

**DOI:** 10.1128/jvi.01487-25

**Published:** 2025-12-09

**Authors:** Qi Yang, Bao Xue, Xianjie Qiu, Kaixin Yang, Jielin Tang, Anqi Zhou, Jingjing Zou, Yuhan Mao, Jiayi Zhong, Yuan Zhou, Wei Zhang, Qiong Zhang, Qingyu Xiao, Wei Tang, Zhiyu Li, Wencai Ye, Gang Zou, Wei Peng, Jinsai Shang, Xi Xu, Yixue Li, Xinwen Chen

**Affiliations:** 1 Guangzhou National Laboratory 612039, Guangzhou International Bio Island, Guangzhou, Guangdong, P.R. China; 2 State Key Laboratory of Respiratory Disease, Guangzhou Medical University 26468, Guangzhou, Guangdong, P.R. China; 3 Chinese Academy of Sciences, Wuhan Institute of Virology 74614, Wuhan, P.R. China; 4 College of Life Sciences, University of Chinese Academy of Sciences, Beijing, P.R. China; 5 State Key Laboratory of Bioactive Molecules and Druggability Assessment, Jinan University 559670, Guangzhou, Guangdong, P.R. China; 6 Department of Medicinal Chemistry, School of Pharmacy, China Pharmaceutical University 56651, Nanjing, P.R. China; 7 Shanghai Ark Biopharmaceutical Co., Ltd 703623 , Shanghai, P.R. China; Loyola University Chicago - Health Sciences Campus, Maywood, Illinois, USA

**Keywords:** respiratory syncytial virus, fusion glycoprotein, lonafarnib, resistant mutations, proteolysis-targeting chimera

## Abstract

Lonafarnib, an oral antiviral that targets the fusion glycoprotein of respiratory syncytial virus (RSV), has demonstrated efficacy *in vitro* and *in vivo*. However, because the RSV has evolved to become resistant to other fusion inhibitors, there is a concern that the same could occur for lonafarnib. Here, we identified resistance to lonafarnib in the RSV A2 strain and a recent clinical isolate, RSV ON1, via *in vitro* selection at scale. Cell‒cell fusion and recombinant live RSV analysis confirmed that the mutations at K394, K399, and T400 of the cysteine-rich region of the fusion protein mediate high-level resistance. Lonafarnib resistance mutations also confer cross-resistance to other fusion inhibitors of clinical interest. All-atom molecular dynamics simulations revealed that these resistance mutations confer reduced stability to the fusion protein, thereby diminishing its binding affinity with lonafarnib. To address this vulnerability proactively and increase the barrier to resistance development, we designed the first potent proteolysis-targeting chimera (PROTAC) fusion protein degrader, compound 0179841, which uses lonafarnib and cereblon as ligands. This PROTAC effectively inhibited RSV replication. Collectively, our findings indicate that RSV develops resistance to lonafarnib in the cysteine-rich region of the fusion protein. This work sheds light on the mechanisms by which RSV evolves resistance to lonafarnib and provides a foundation for the rational design of antivirals aimed at preventing resistance.

**IMPORTANCE:**

Respiratory syncytial virus (RSV) infection poses a substantial public health challenge. Resistance to several potent fusion inhibitors, which are currently in various stages of clinical development, can readily emerge. Through a drug repurposing screen, we identified lonafarnib as an RSV fusion inhibitor; however, concerns exist regarding the potential development of resistance. Here, large-scale *in vitro* selection experiments revealed specific mutations within the highly conserved cysteine-rich region of the fusion (F) protein that confer high-level lonafarnib resistance across diverse RSV strains. These resistance mutations also confer cross-resistance to other clinical-stage fusion inhibitors. Mechanistic investigations demonstrated that these mutations reduce F protein stability, thereby diminishing the binding affinity of lonafarnib. As a proof of concept for an alternative antiviral strategy, we rationally designed the first potent proteolysis-targeting chimera (PROTAC) F protein degrader, compound 0179841, by utilizing lonafarnib and cereblon ligands. This novel antiviral agent effectively inhibits RSV infection by inducing degradation of the F protein. This work elucidates the molecular basis of RSV resistance to lonafarnib and establishes a strategy for developing next-generation antivirals aimed at preempting resistance.

## INTRODUCTION

Respiratory syncytial virus (RSV) is a common cause of acute lower respiratory tract infection in infants, older adults, and immunocompromised individuals (1, 2). Despite the clinical benefits of three approved RSV vaccines (Arexvy (3) [GSK]; Abrysvo (4) [Pfizer]; or mResvia (5) [Moderna]) and two monoclonal antibodies (palivizumab (6) [AstraZeneca] and nirsevimab (7) [AstraZeneca, Sanofi]), treatment remains limited to high-risk people, with approval in only a few countries and regions. Arexvy, Abrysvo, and mResvia are available for adults; however, no vaccines are approved for the prevention of RSV infection in infants or children (8). The Food and Drug Administration recently suspended all clinical trials of RSV vaccines in infants and young children following reports of severe illness in infants vaccinated with Moderna’s mRNA-based RSV vaccine (9). Palivizumab and nirsevimab are approved for the prevention but not the treatment of RSV disease (10).

There are currently no approved RSV-specific therapeutic small molecules available, but multiple clinical-stage small molecules, including fusion inhibitors targeting the viral fusion glycoprotein (F) (11–15) and replication inhibitors targeting the viral nucleoprotein (N) (16) or large polymerase subunit (L) (16–18), are currently available as drug candidates for treating RSV infections. These three viral proteins are essential for the viral life cycle and are well conserved among Orthopneumoviruses (19, 20). Nevertheless, the development of mutational escape and therapeutic resistance is still common for RSV (14, 15, 21–28), which is a particular concern in immunocompromised individuals.

The RSV F protein plays a critical role in mediating viral entry into host cells (20, 29) and induces the formation of syncytia between infected cells and neighboring uninfected cells (30, 31). Inhibition of viral entry and spread by blocking the exchange of a metastable state (prefusion) with a stable state (postfusion) of the RSV F protein during the fusion process has emerged as a promising treatment option for patients infected with RSV (19, 32). Currently, GS-5806 (14), AK0529 (15), RV521 (12), and JNJ-53718678 (13) are several structurally heterogeneous and potent fusion inhibitors that are being clinically developed for RSV infection treatment. However, all of these inhibitors have the potential to induce the emergence of resistant mutants both *in vitro* and *in vivo* (14, 15, 21, 22, 24, 26), as demonstrated by resistance-associated substitutions for GS-5806 (e.g., T400A, D486N) (21), JNJ-53718678 (e.g., L141W, D489Y) (22), AK0529 (e.g., D486N, D489A/V/Y) (24), and RV521 (e.g., D489Y) (26) *in vitro*, and the most frequent substitutions (e.g., F140I, L141F/W, S398L, K399N, T400A/I, D486N, or F488L) in the F protein to GS-5806 or AK0529 have already been identified in real-world cases (14, 15). Among the known mutations, D486N and D489Y have shown cross-resistance to various fusion inhibitors, and the emergence of both mutants is a potential concern for human health (33). Thus, new therapeutic options are still needed.

Lonafarnib, an orally active farnesyltransferase inhibitor, has demonstrated high therapeutic efficacy for Hutchinson‒Gilford progeria syndrome (HGPS) (34) and is a phase III candidate for hepatitis delta virus (HDV) therapy (35). Recently, drug repurposing screens conducted by our team and another research group identified lonafarnib as a potent RSV fusion inhibitor (36, 37). In a murine infection model, oral administration of lonafarnib resulted in a dose-dependent reduction in the RSV load and alleviation of lung damage. Thus, the repurposing of lonafarnib has significant potential in combating RSV infection and may offer a more accessible treatment option for patients with HGPS, HDV, or cancer comorbidities. However, whether RSV can evolve resistance mechanisms similar to those observed with other fusion inhibitors remains a concern.

The emergence of novel viruses and drug-resistant strains highlights the urgent need for innovative antiviral therapies (38). Targeted protein degradation (TPD) represents an innovative drug discovery strategy that eliminates proteins of interest (POIs) by harnessing the intracellular ubiquitin-proteasome system (UPS), macroautophagy, and endolysosomal pathways (39). Proteolysis-targeting chimera (PROTAC) molecules serve as classic representatives of TPD and typically comprise three key components: a ligand for the POI, a linker, and a ligand for the E3 ligase. By bridging the POI and the E3 ligase, PROTACs facilitate the ubiquitination of the POI, thereby triggering its degradation through the proteasome system (40). In recent years, substantial research efforts have been devoted to the development of antiviral drugs that target a variety of viruses via PROTAC technology (such as hepatitis B virus [41], hepatitis C virus [42], influenza virus [43, 44], and SARS-CoV-2 [45, 46]). However, the potential of using PROTAC modalities to target the F protein for degradation and thereby inhibit RSV infection remains largely unexplored.

Here, we identified the conserved cysteine-rich region of the RSV F protein as the critical site associated with resistance to lonafarnib *in vitro*. To investigate the underlying molecular mechanisms of RSV resistance to lonafarnib, we conducted surface plasmon resonance (SPR) and all-atom molecular dynamics (MD) simulation assays for each resistance mutation in the presence of lonafarnib. Furthermore, we applied PROTAC approaches to develop a lonafarnib-based virus-specific antiviral degrader that targets the viral F protein.

## RESULTS

### Screening for RSV resistance to lonafarnib

**Fig 1:**
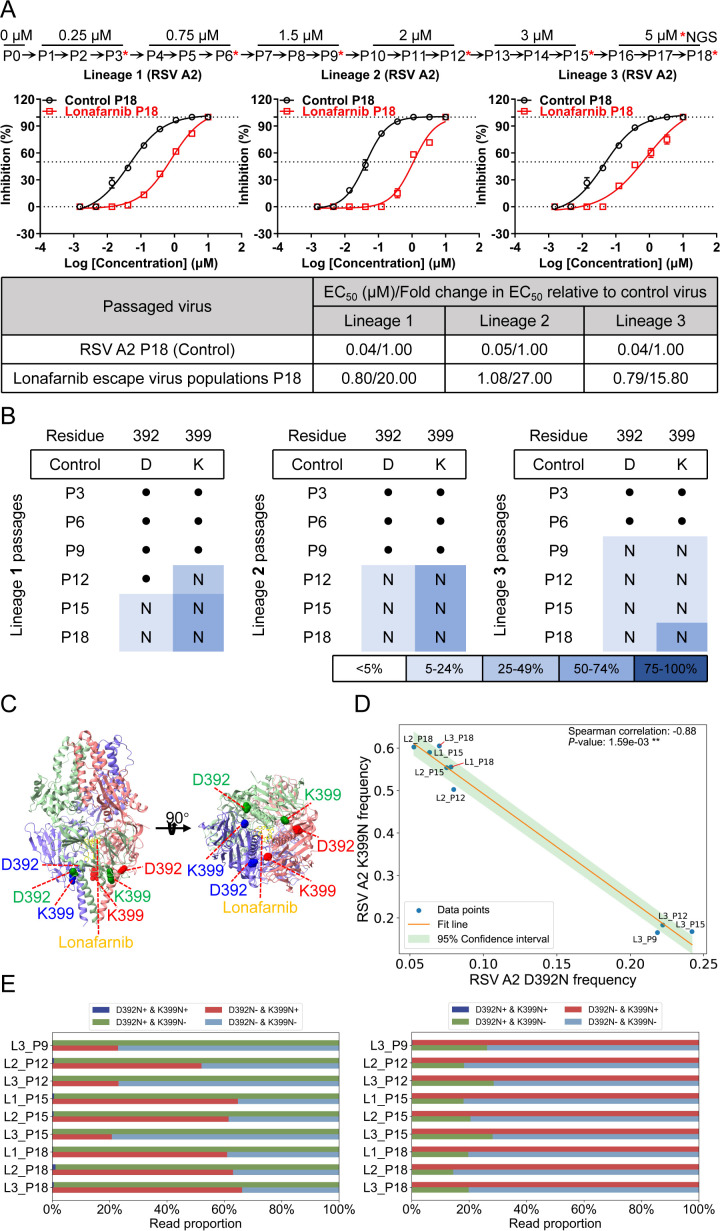
Identification of the resistance of the RSV A2 strain to lonafarnib. (**A**) Passaging scheme. HEp-2 cells were infected in triplicate with RSV A2 and passaged into fresh cells every 2 days for 18 passages, and the concentration of lonafarnib was increased every three passages. Changes in the EC_50_ during passaging of RSV A2 with lonafarnib. Validation of lonafarnib resistance in the indicated passages from each of the three lineages (1 [left], 2 [middle], and 3 [right]). The graphs shown are the mean values of three independent experiments. (**B**) Mutations in the F protein were found in the indicated passages from each lineage. The dots indicate the control virus at that residue. Mutations are shaded according to frequency (see Table S1 for exact frequencies). (**C**) Residues mutated with passaging are overlaid onto the DS-Cav1 structure with lonafarnib bound. The Cα of each mutated residue is denoted by a colored sphere. The DS-Cav1-lonafarnib complex was downloaded from PDB under accession code 8KG5. (**D**) Spearman correlation analysis between D392N and K399N mutations in the RSV A2 F protein. Correlation analysis of D392N and K399N mutations with frequencies > 5% between all nine samples. (**E**) Linkage relationship between D392N and K399N mutations. The reads of the D392N and K399N mutations were extracted from nine samples simultaneously with two mutations. The number of reads with only the D392N mutation and only the K399N mutation, as well as the number of reads with neither mutation and the simultaneous appearance of two mutations, were counted via a contingency table, and a χ^2^ test for independence on this contingency table was performed. The proportions of K399N mutations in the D392 control and D392N mutation reads (left), and the proportions of D392N mutations in the K399 control and K399N mutation reads (right) were calculated.

To identify resistance mutations against lonafarnib, the RSV A2 strain was passaged in HEp-2 cells under increasing drug concentrations, and viruses from every third passage were subjected to next-generation sequencing (NGS) (Fig. 1A and Methods). Triplicate lineages developed high-level resistance after 18 passages, exhibiting a mean 20.9-fold increase in half-maximal effective concentration (EC_50_) values compared with those of the control virus (Fig. 1A). NGS revealed that the D392N and K399N mutations appeared in the F gene of the RSV A2 strain in all three lineages, the K399N mutation of which occurred in a stepwise manner (Fig. 1B; Table S1). The D392N and K399N mutations are located in the 392–401 microdomain of the F protein and in immediate proximity to the lonafarnib-binding microdomain (486–489 residues) in prefusion F (Fig. 1C) (36, 37). Correlation analysis of the mutation frequencies with >5% mutation across all nine samples revealed a significant negative correlation (Spearman coefficient of −0.88, *P <* 0.01) (Fig. 1D). χ^2^ testing of paired-read distributions confirmed non-independence, indicating mutual exclusivity (Fig. 1E).

**Fig 2:**
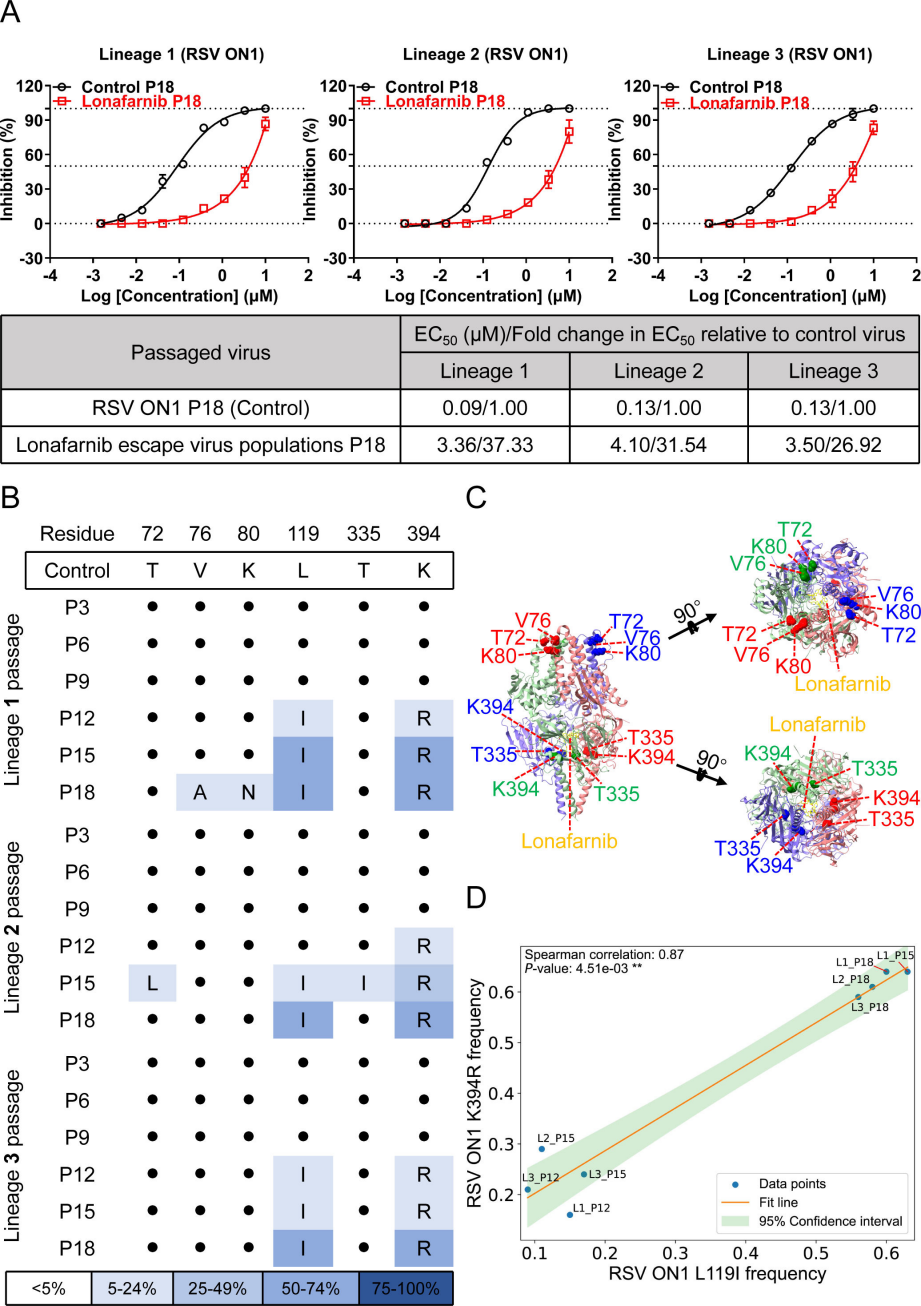
Identification of the resistance of the RSV ON1 strain to lonafarnib. (**A**) HEp-2 cells were infected in triplicate with RSV ON1 and passaged into fresh cells every 2  days for 18 passages, and the concentration of lonafarnib was increased every three passages. Changes in the EC_50_ for the indicated passages from each of the three lineages were observed. Validation of lonafarnib resistance in the indicated passages from each of the three lineages (1 [left], 2 [middle], and 3 [right]) was performed. The graphs shown are the mean values of three independent experiments. (**B**) Mutations in the F protein were found in the indicated passages from each lineage. The dots indicate the control virus at that residue. Mutations are shaded according to frequency (see Table S2 for exact frequencies). (**C**) Residues mutated with passaging are overlaid onto the DS-Cav1 structure with lonafarnib bound. The Cα of each mutated residue is denoted by a colored sphere. The DS-Cav1-lonafarnib complex was downloaded from PDB under accession code 8KG5. (**D**) Spearman correlation analysis between L119I and K394R mutations of the RSV ON1 F protein. Correlation analysis of L119I and K394R mutations with frequencies > 5% across all 8 samples.

Parallel passaging of the clinical isolate RSV ON1 (18 passages, with triplicate lineages) in the presence of increasing concentrations of lonafanib yielded EC_50_ increases ranging from 26.9-fold to 37.3-fold (Fig. 2A). Six mutations in the RSV F gene were identified: T72L, V76A, K80N, L119I, T335I, and K394R (Fig. 2B; Table S2). Four mutations (T72L, V76A, K80N, and T335I) presented a low prevalence (<10% mean frequency): T72L (7.2% in lineage 2), V76A (8.0% in lineage 1), K80N (7.8% in lineage 1), and T335I (5.1% in lineage 2). These four mutations occurred in a non-stepwise manner. In contrast, L119I and K394R were conserved across lineages and detected in passages 15–18. The amino acid change at position 119 is in the 27-amino-acid peptide (p27), which is removed by furin during the post-translational processing of the F protein (47, 48). The 394th amino acid residue is located in the 392–401 microdomain of the F protein and is in immediate proximity to the lonafarnib-binding microdomain (486–489 residues) in prefusion F (Fig. 2C) (36, 37). Owing to the low mutation frequency and skip-like occurrence of these four sites (T72L, V76A, K80N, and T335I), we only conducted correlation analysis on L119I and K394R mutations with frequencies > 5% between all eight samples, which showed a significant positive correlation (Spearman coefficient of 0.87, *P* < 0.01) (Fig. 2D). Physical distance precluded read-level independence analysis between the L119I and K394R mutations.

Overall, selection at the scale revealed that RSV can lead to the development of lonafarnib resistance. The differences in mutation sites could arise in the RSV A2 and ON1 strains, and all these mutations were not situated in the lonafarnib-binding sites; rather, several mutations in the 392–401 microdomain of the RSV F protein were preferred.

### Characterization of lonafarnib-resistant mutants

**Fig 3:**
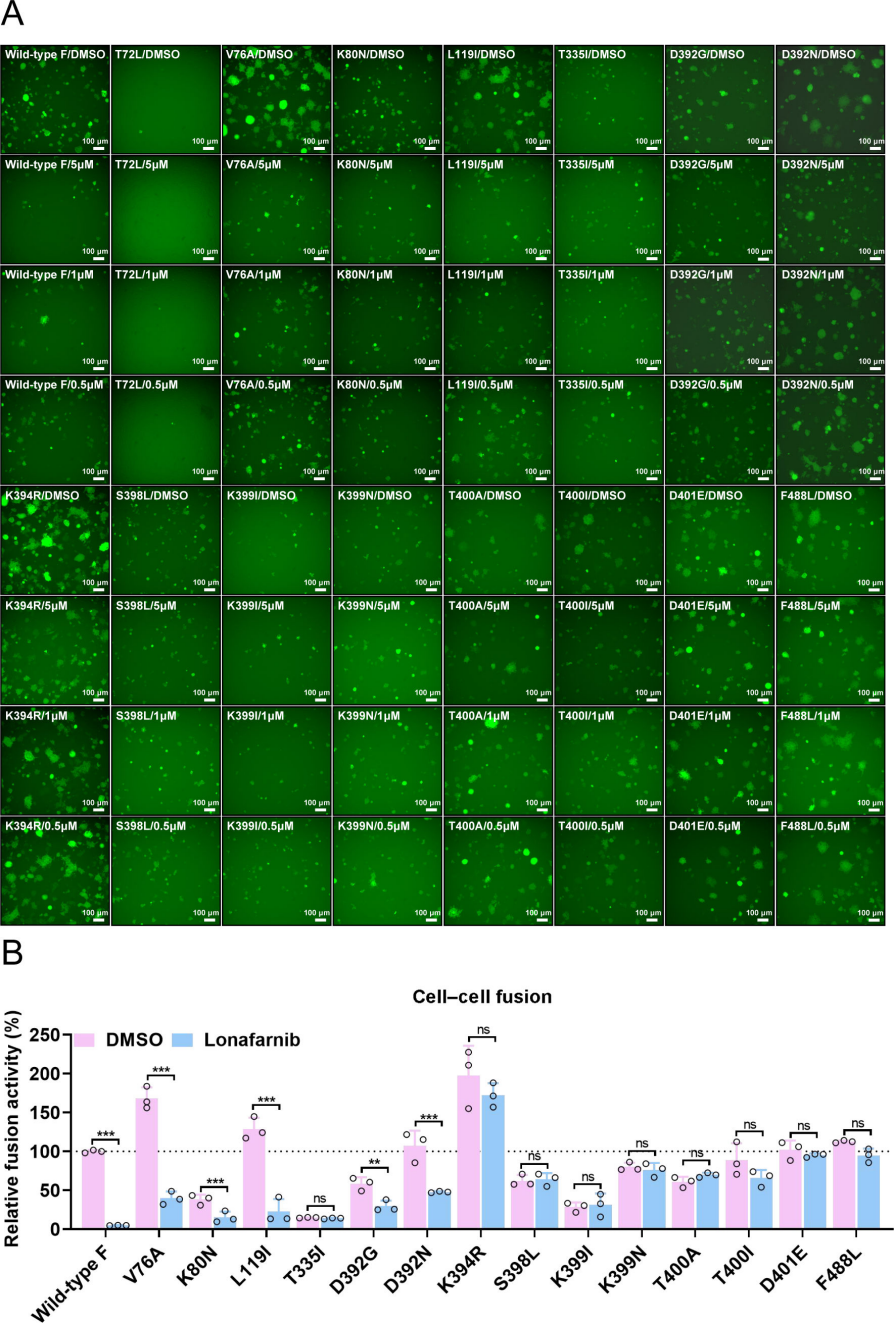
Validation of the resistance of the identified mutations to lonafarnib via a cell‒cell fusion assay. (**A**) Effects of lonafarnib on wild-type F- or F mutation-mediated cell‒cell fusion. All mutations identified in this study (T72L, V76A, K80N, L119I, T335I, D392N, K394R, and K399N) and other resistance-associated substitutions (D392G/N, K394R, S398L, K399I/N, T400A/I, and D401E) reported for fusion inhibitors in the 392–401 microdomain were constructed. F488 serves as a binding site for lonafarnib and acts as an experimental positive control. HEK293T GFP-split cells were transfected with plasmids expressing wild-type F or F mutants and treated with 0.1% DMSO or lonafarnib (5, 1, and 0.5 µM). The cell‒cell fusion activity was determined by GFP expression 24 h post-transfection. Images represent three biological replicates comprising nine fields each. (**B**) Quantification of cell‒cell fusion activity was performed via ImageJ analysis software. For statistical analysis, nonparametric Mann‒Whitney tests were used for comparisons. The results are representative of three random experiments. The error bars indicate the means ± SDs of three technical replicates. **P < 0.01, ***P < 0.001; ns, not significant.

To comprehensively investigate which mutations are responsible for lonafarnib resistance, we constructed eight mutations identified in this study (T72L, V76A, K80N, L119I, T335I, D392N, K394R, and K399N) and other fusion inhibitors reported resistance-associated substitutions (D392G/N, K394R, S398L, K399I/N, T400A/I, and D401E) in the 392–401 microdomain (15, 21, 36, 49–52). Using a GFP-split fusion model for cell‒cell fusion mediated by the RSV F protein (Fig. S1), we observed that the substitutions in RSV F resulted in its loss (T72L), decrease (K80N, T335I, and K399I), or increase (V76A, L119I, and K394R) in membrane fusion ability (Fig. 3A and B). Other substitutions (D392G/N, S398L, K399N, T400A/I, D401E, and F488L) of RSV F have the same ability to induce fusion as the wild-type F protein does. F488 serves as a binding site for lonafarnib (37) and acts as an experimental positive control. F488L alteration decreased drug binding and resulted in a loss of inhibitory activity. Compared with wild-type F, lonafarnib significantly inhibited V76A, K80N, L119I, and D392G/N mutant-induced cell‒cell fusion activity, suggesting that alterations at these sites are largely not responsible for lonafarnib resistance (Fig. 3A and B). Lonafarnib did not or only slightly inhibit the fusion activity induced by T335I, S398L, K394R, K399I/N, T400A/I, and D401E, indicating that these mutations in the cysteine-rich region might lead to the resistance of RSV to lonafarnib.

Next, we investigated mutations in recombinant live RSV carrying single F protein mutations. Seven mutant viruses (V127G, L141F, K394R, K399N, T400A, T400I, and F488L) were used in this study, with V127G and L141F mutant viruses used as the experimental negative controls and F488L as the positive control. The infectious titer produced by HEp-2 cells infected with wild-type RSV increased faster than did the titer produced by the viruses with mutations (Fig. S2A), and after 90 h, the titer of the wild-type virus was almost 10-fold greater than the titer achieved by viruses with mutations except for V127G (Fig. S2A). Among the individual mutants tested against lonafarnib, the infection of the wild-type and V127G mutant viruses was effectively inhibited; L141F showed only minimal resistance (~1.7-fold to 3.3-fold); K399N (~9.8-fold increase in the EC_50_ and ~8.2-fold increase in the EC_90_), and F488L (~15.2-fold increase in the EC_50_ and ~10-fold increase in the EC_90_) presented high-level resistance relative to that of the wild-type virus; the increased resistance caused by T400A (~33-fold increase in the EC_50_ and ~24-fold increase in the EC_90_) was approximately equivalent to that caused by T400I; K394R (~111.5-fold increase in the EC_50_ and ~79.2-fold increase in the EC_90_) was the most resistant (Table 1; Fig. S2B). Together, these results indicate that the K394R, K399N, and T400A/I mutations in the cysteine-rich region of the F protein favor RSV resistance to lonafarnib.

**TABLE 1:** EC_50_ and EC_90_ of lonafarnib against wild-type RSV and mutant strains^*a*^

RSV strain	EC_50_ (μM)	Fold change in EC_50_ relative to wild-type	EC_90_ (μM)	Fold change in EC_90_ relative to wild-type
Wild-type	0.046 ± 0.010	1.0	0.190 ± 0.120	1.0
V127G	0.044 ± 0.005	0.964	0.177 ± 0.038	0.935
L141F	0.079 ± 0.019	1.717	0.631 ± 0.125	3.326
K394R	5.127 ± 1.689	111.464	15.025 ± 7.331	79.202
K399N	0.450 ± 0.032	9.786	1.552 ± 0.277	8.183
T400A	1.522 ± 0.127	33.087	4.640 ± 0.440	24.458
T400I	1.506 ± 0.184	32.746	4.611 ± 0.663	24.308
F488L	0.700 ± 0.069	15.228	1.900 ± 0.160	9.992

^*a*^ V127G and L141F mutant viruses were used as experimental negative controls; F488L was used as the positive control.

Furthermore, we investigated mutant viruses (K394R, K399N, and T400A) against other clinically relevant fusion inhibitors (e.g., GS-5806 (14), AK0529 (15), RV521 (12), and JNJ-53718678 (13)) for cross-resistance. All three of these mutant viruses exhibited considerable resistance to the above four fusion inhibitors, but K399N resulted in only slight resistance to RV521 (Table 2; Fig. S3). Selection for lonafarnib resistance can clearly yield mutations that confer cross-resistance to other inhibitors of clinical interest as well.

**TABLE 2:** Antiviral activities of RSV F inhibitors against wild-type RSV or RSV variants

Inhibitor	EC_50_ (nM)		Fold resistance^*a*^	EC_50_ (nM)	Fold resistance	EC_50_ (nM)	Fold resistance
	RSV	RSV K394R		RSV K399N		RSV T400A	
Lonafarnib	45.6	3,839.0	84.3	405.6	8.9	1,117.0	24.5
GS-5806	0.1	192.5	1,673.9	4.4	38.3	28.8	250.1
AK0529	1.3	6,553.0	4,983.3	2,253.0	1,713.3	9,851.0	7,491.3
RV521	0.1	178.9	2,462.8	0.4	5.4	12.6	173.6
JNJ-53718678	0.7	10,130.0	15,582.2	248.5	382.2	11,650.0	17,920.3

^*a*^ Fold resistance is the fold change in the EC_50_ relative to that of wild-type RSV.

### Mechanism of lonafarnib resistance

**Fig 4:**
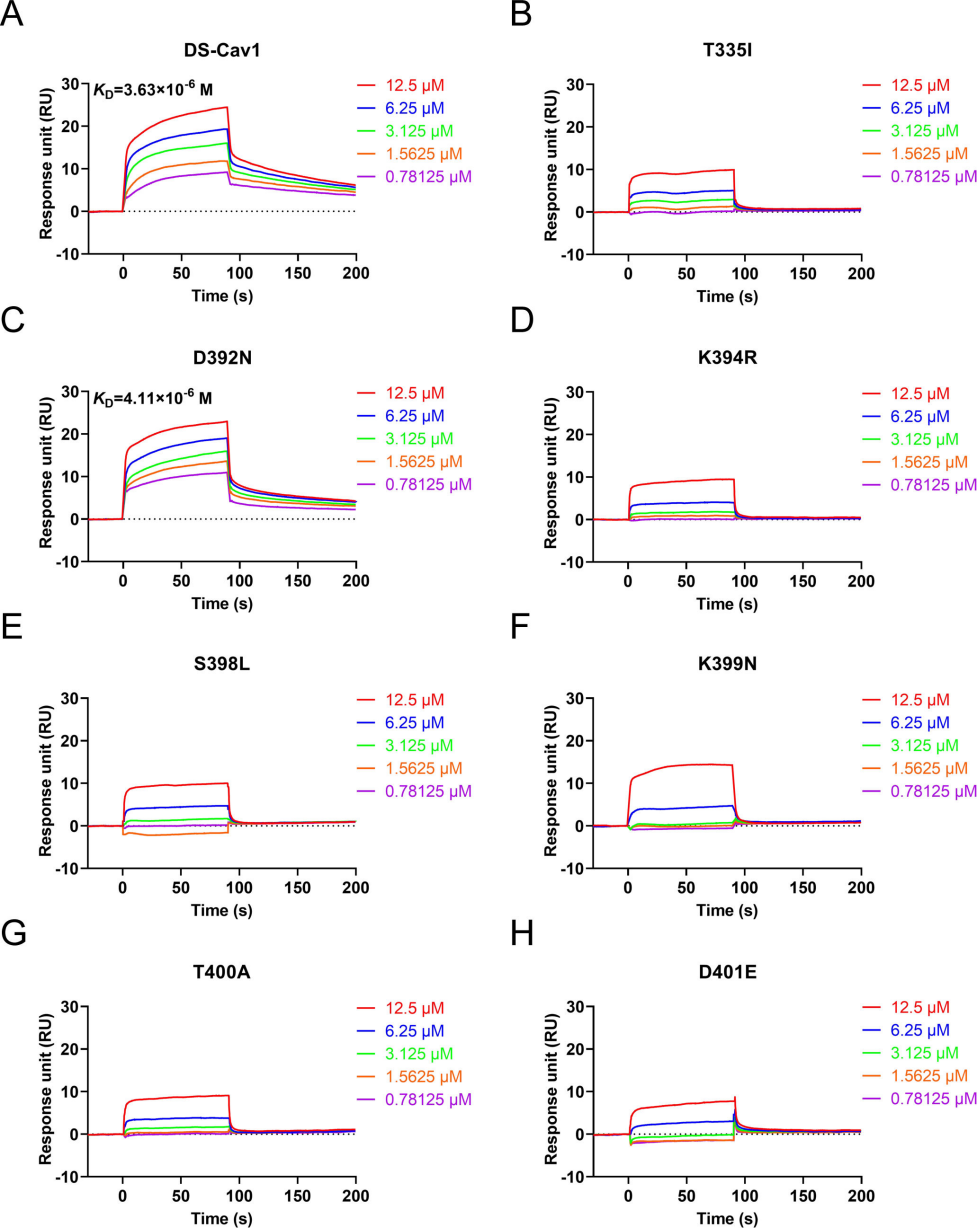
Binding affinity of prefusion-stabilized RSV F variant DS-Cav1 and its mutants for lonafarnib. (**A–H**) BIAcore diagrams of DS-Cav1, T335I, D392N, K394R, S398L, K399N, T400A, and D401E binding to lonafarnib. These mutations D392G/N, K399I/N, and T400A/I exhibit no significant difference in their anti-fusion capabilities in cell‒cell fusion assays; thus, only one mutation at the same site was selected for SPR assays. The graphs shown are representative of three independent experiments.

**Fig 5:**
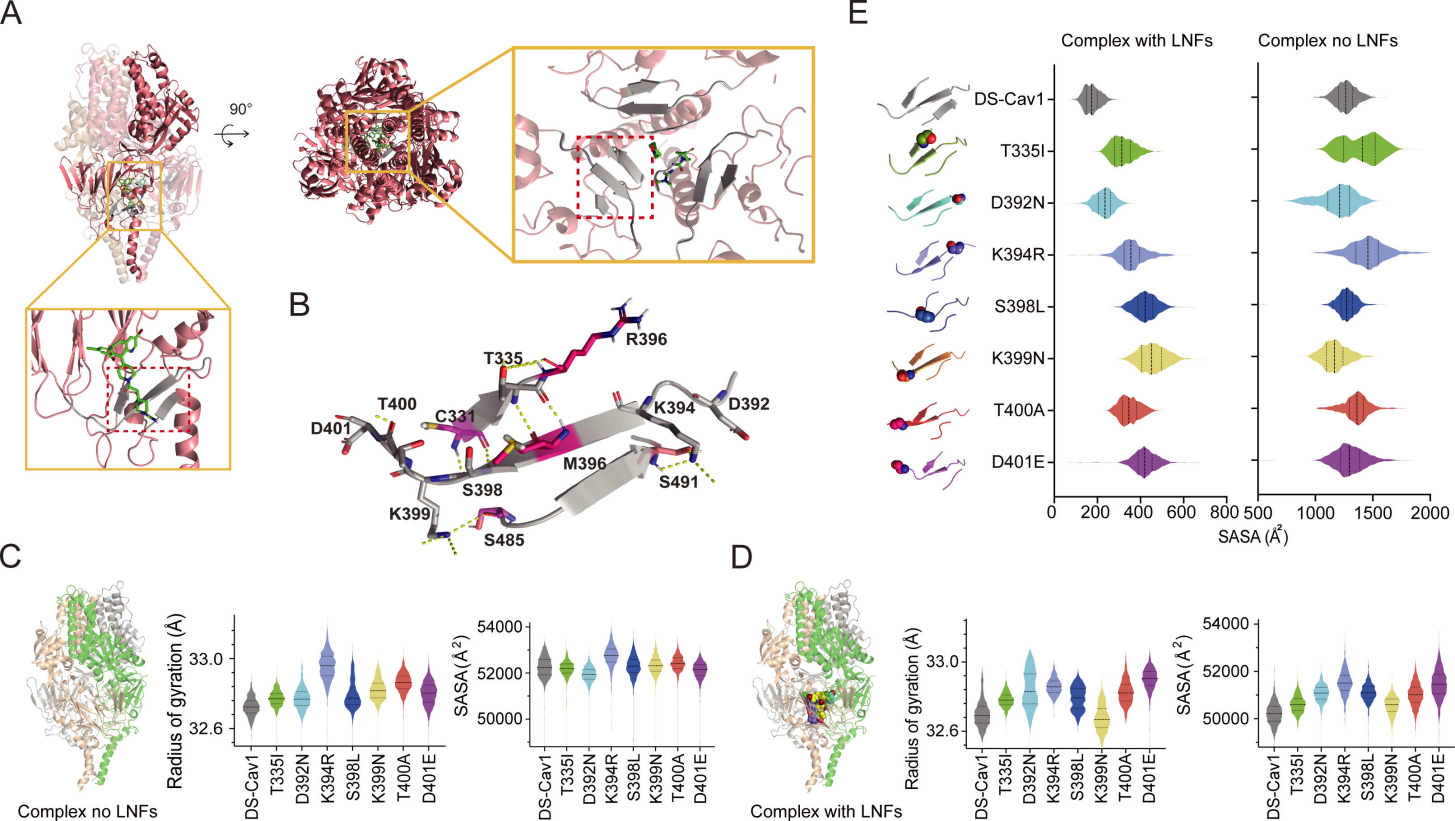
All-atom dynamic simulations of DS-Cav1 and seven mutant residues (T335I, D392N, K394R, S398L, K399N, T400A, and D401E) of the RSV F protein. (**A**) Schematic view of the main cluster of the molecular dynamics simulation. The yellow rectangular box outlines the central cavity region, and the red dotted-line box outlines the regions of the three stable β-sheet residues (**B**). Complex MD models without (**C**) or with (**D**) lonafarnibs (LNFs). The radius of gyration (Rg) directly measures protein stability, showing its folding state and compactness. The solvent-accessible surface area (SASA) reflects stability by indicating the protection of the hydrophobic core. Both parameters enable a comprehensive assessment of protein stability. (**E**) Statistical analysis of the SASA values of the three stable beta-sheet residues with DS-Cav1 and seven different cases of mutant residues in the RSV F complex with or without LNFs.

To investigate the mechanisms underlying RSV resistance to lonafarnib in the cysteine-rich region of the F protein, we expressed prefusion-stabilized RSV F variant DS-Cav1 (19) and its mutants (T335I, D392N, K394R, S398L, K399N, T400A, and D401E) in HEK293F cells (Fig. S4A and B). SPR analysis revealed that the mutants (T335I, K394R, S398L, K399N, T400A, and D401E) significantly decreased the binding affinity for lonafarnib (Fig. 4B and D through H). Compared with DS-Cav1, the D392N mutant retained the ability to bind to lonafarnib but presented slightly lower affinities (Fig. 4A and C). Next, all-atom MD simulations were conducted on DS-Cav1 and these mutants. The simulations were executed for 500 ns and replicated three times (Fig. S5) under two different conditions, where the prefusion F trimer complex was present in the absence or presence of lonafarnib. These residues are located around the central cavity, which includes a cysteine-rich region and heptad repeat B (Fig. 5A and B). The DS-Cav1 variant presented three stable β-sheet conformations in the main cluster structure (Fig. 5A and B), and these β-sheets formed at residues 331–335, 394–398, and 486–488 in the DS-Cav1 cluster structure. As shown, these residues engage in polar contacts: C331 with S398, T335 with M396, K394 with S491, and K399 with S485 (Fig. 5B). The formamide group of the lonafarnib chemical structure is crucial for binding by providing polar interactions, which form hydrogen bonds with residues T397, S398, D486, and E487 (37). The radius of gyration (Rg) directly measures protein stability, showing its folding state and compactness. The solvent-accessible surface area (SASA) reflects stability by indicating the protection of the hydrophobic core. Both parameters enable a comprehensive assessment of protein stability. MD simulations revealed that the DS-Cav1 and D392N mutations were the most stable among all the simulation cases (Fig. 5C). Conversely, the K394R mutation resulted in the highest values of both Rg and SASA, indicating a decrease in stability compared with those of DS-Cav1 and other mutants. Notably, even in the presence of lonafarnib, DS-Cav1 still maintained its status as the most stable (Fig. 5D). Similarly, K394R also presented the highest Rg and SASA values, indicating its relatively lower stability than the other mutations.

To investigate the effects of lonafarnib on various mutations, we analyzed the SASA values of residues surrounding the central cavity, which are residues 333–335, 392–401, and 486–488. The results demonstrated that the DS-Cav1 and D392N mutants presented increased binding affinity for lonafarnib (Fig. 5E). In contrast, other mutations significantly weakened the interaction between the F protein and lonafarnib. Notably, in the RSV F complex model with the K399N mutation, relatively low Rg and SASA values were observed (Fig. 5D), suggesting that lonafarnib may still bind to this mutation. However, the central cavity area of the K399N mutation appears less stable than that of the DS-Cav1 and D392N mutations (Fig. 5E). These findings are consistent with previous experimental data (Fig. 4), validating the role of mutations in modulating the lonafarnib interaction within the molecular mechanisms.

### Compound 0179841-induced degradation of the F protein confers an anti-RSV effect

**Fig 6:**
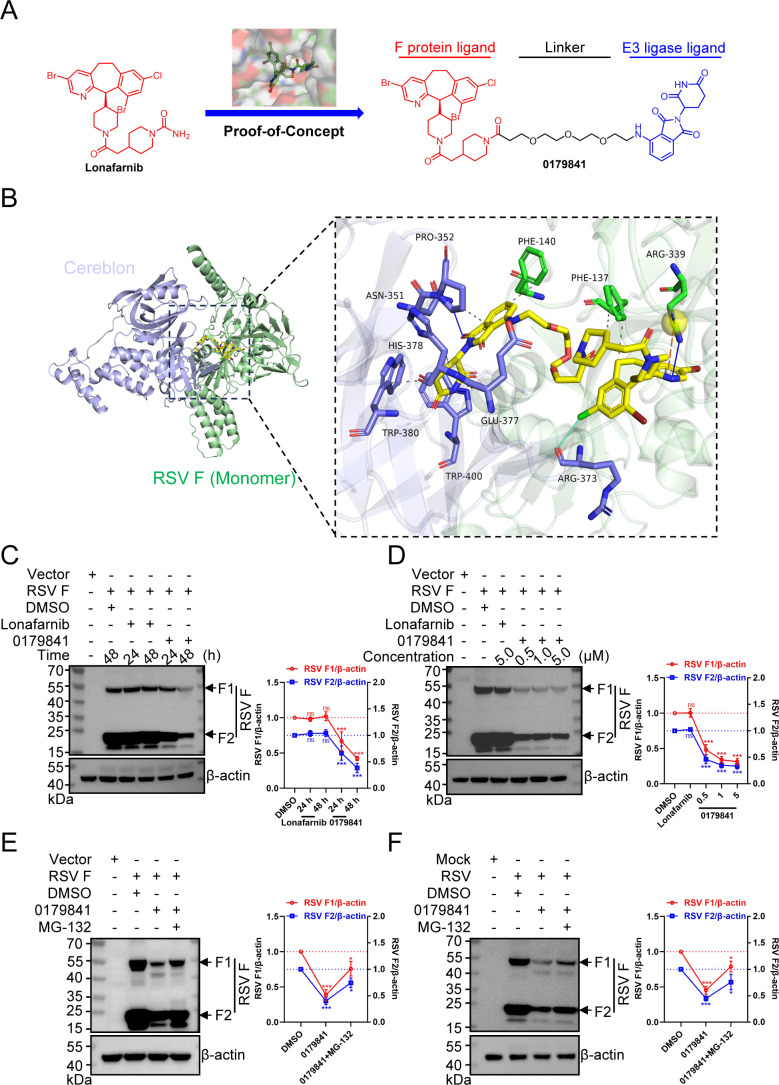
The PROTAC 0179841 depletes the RSV F protein. (**A**) Structure of virus-specific and host-directed antivirals utilizing targeted protein degradation, comprising a lonafarnib-derived F protein ligand (red), a PEG-based flexible linker (black), and a cereblon-targeting E3 ubiquitin ligase ligand (blue). (**B**) Docking model of the binding of compound 0179841 to the RSV F monomer and cereblon. The RSV F monomer is depicted as green sticks, the cereblon is depicted as blue sticks, and compound 0179841 is depicted as yellow sticks. (**C–E**) HEK293T cells were transfected with F plasmids, and the cell lysates were analyzed via western blotting with antibodies against RSV F or β-actin (left) and densitometric analysis (right). (**C**) The transfected cells were treated with 0.1% DMSO, 1 µM lonafarnib, or compound 0179841 for the indicated periods post-transfection. (**D**) The transfected cells were treated with 0.1% DMSO or the indicated compounds at the indicated concentrations for 48 h. (**E**) The transfected cells were treated with 0.1% DMSO or 1 µM compound 0179841 for 48 h, and the cells were then treated with either 0.1% DMSO or 20 µM MG-132 for 8 h before sample collection. (**F**) HEp-2 cells were infected with RSV (MOI = 10) and subsequently treated with either 0.1% DMSO or 1 µM compound 0179841 for 18 h. Prior to sample collection, the cells were further treated with either 0.1% DMSO or 20 µM MG-132 for 8 h. The cell lysates were then analyzed by western blotting using antibodies against RSV F and β-actin (left) and densitometric analysis (right). The graphs shown are representative of three independent experiments, and the error bars indicate the means ± SDs derived from these three independent experiments. *P < 0.05, ***P < 0.001; ns, not significant (one-way ANOVA with Dunnett’s post hoc test).

The resistance mutations in the central cavity region of the RSV F protein confer resistance by structurally destabilizing the protein and reducing the binding affinity of lonafarnib. Crucially, the long-range impact of these mutations on the *β*-sheet network and hydrophobic cavity conformation underscores how current fusion inhibitors remain susceptible to allosteric resistance mechanisms. As most small-molecule candidates targeting the RSV F protein pose a considerable risk of inducing drug resistance mutations, these findings highlight the pressing need for new approaches to address this vulnerability proactively and overcome the barrier to resistance development. Here, we applied the PROTAC approach to develop a lonafarnib-based antiviral degrader, 0179841, that targets the RSV F protein. The molecule 0179841 anchors to the hydrophobic domain of the RSV F protein through its lonafarnib module and recruits the E3 ubiquitin ligase complex via the cereblon ligand, thereby enabling ubiquitination-dependent degradation of the target (Fig. 6A; see synthetic details in Fig. S6 and S7). Computational docking predicted the binding conformation between PROTAC 0179841 and both the RSV F protein and the cereblon protein. The best binding poses were identified on the basis of geometric and energy matching, yielding a binding score of −11.38. The results showed that compound 0179841 interacts directly with three residues (Phe137, Phe140, and Arg339) in the monomeric F protein and two residues (Asn351 and Trp380) in the cereblon protein (Fig. 6B). Four sets of experiments were conducted to assess the selective degradation and inhibitory effects of compound 0179841 on the RSV F protein. In HEK293T cells expressing the RSV F protein, we measured the protein levels of F with and without compound 0179841 treatment. We found that compound 0179841-induced degradation of the F protein is time- and concentration-dependent (Fig. 6C and D), which appears to occur through proteasomal degradation pathways (Fig. 6E), but lonafarnib did not affect F protein levels (Fig. 6C and D). Notably, compound 0179841 also induced significant degradation of the F protein during RSV infection (Fig. 6F).

**Fig 7:**
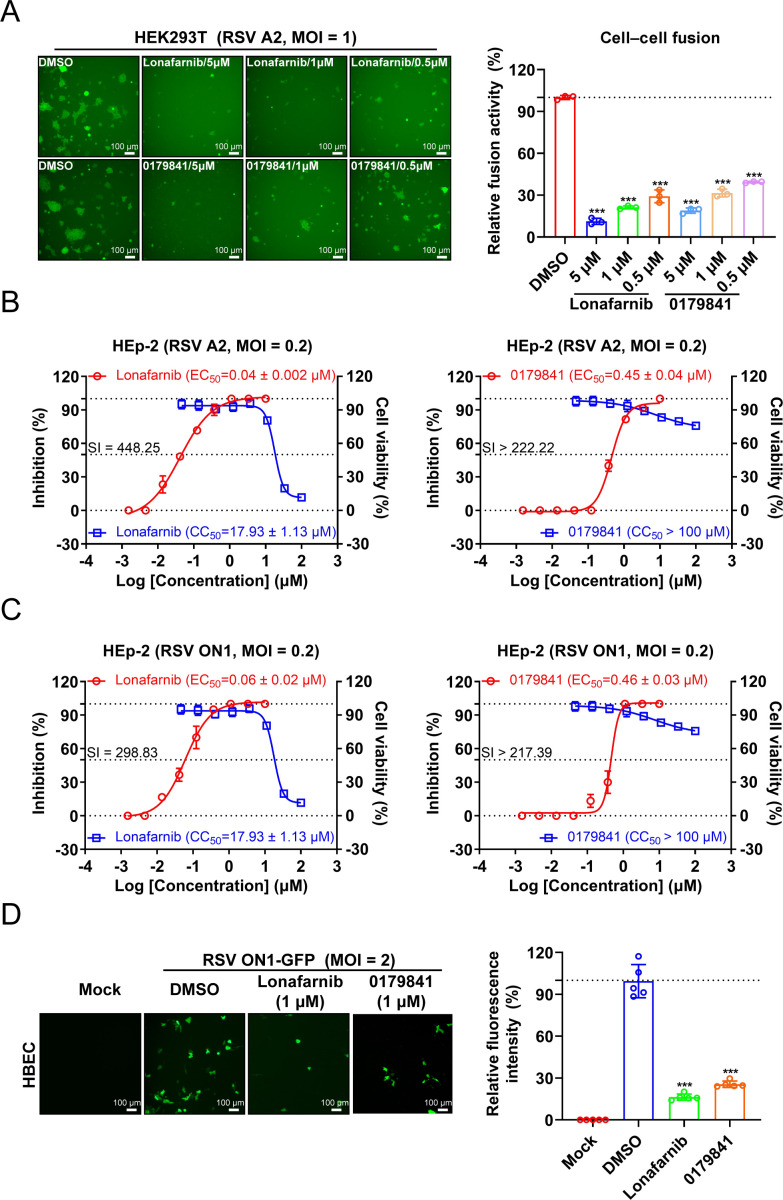
*In vitro* anti-RSV activity of compound 0179841. (**A**) Effect of compound 0179841 on RSV-mediated cell‒cell fusion. HEK293T GFP-split cells were infected with RSV A2 (MOI = 1) and treated with lonafarnib or compound 0179841 at the indicated concentrations (5, 1, and 0.5 µM) or with 0.1% DMSO as a control. Representative images of cell‒cell fusion at 24 h post-infection are shown (left). The quantification of cell‒cell fusion activity according to the GFP intensity in three random fields per experiment was performed via ImageJ analysis software, and the data were normalized to the intensity in the DMSO condition (right). (**B, C**) HEp-2 cells were infected with the RSV A2 (**B**) or ON1 (**C**) strain (MOI = 0.2) and treated with the indicated concentrations of the indicated compounds for 72 h post-infection. The inhibitory effects of the indicated compounds on RSV were quantified via FFA. The EC_50_ represents the concentration required to inhibit RSV infection by 50%. The cytotoxicity of the indicated compounds to HEp-2 cells was measured via cell viability assays. The selectivity index (SI) = CC_50_/EC_50_ was calculated. (**D**) Effects of compound 0179841 on RSV infection in primary human bronchial epithelial cells (HBECs). HBECs were infected at an MOI of 2 with the RSV ON1-GFP strain and treated with either 1 µM lonafarnib or compound 0179841. The infected cells were subsequently visualized at 72 h post-infection (left). The GFP intensity in five randomly selected fields per experiment was measured via ImageJ analysis software and normalized to the intensity in the DMSO condition (right). The graphs shown are representative of three independent experiments. The error bars represent the means ± SDs from three technical replicates in panel (**A**) and five technical replicates in panel (**D**). The differences between the experimental and DMSO groups were determined by Student’s *t*-test for comparing two groups of data (***P < 0.001).

Next, using the GFP-split fusion model for cell‒cell fusion mediated by RSV, we observed that the compound 0179841 significantly inhibited RSV-induced cell‒cell fusion activity (Fig. 7A). Compound 0179841 also effectively inhibited the replication of the RSV A2 and ON1 strains in HEp-2 cells without affecting cell viability, with EC_50_ values of 0.45 ± 0.04 µM and 0.46 ± 0.03 µM, respectively (Fig. 7B and C). The selectivity index (SI) of compound 0179841 exceeds 217.39 in HEp-2 cells, indicating its safety at the cellular level. Furthermore, we evaluated the *in vitro* antiviral efficacy of compound 0179841 against RSV ON1-GFP in primary human bronchial epithelial cells (HBECs). Treatment with compound 0179841 resulted in a significant decrease in the RSV infection level, as evidenced by marked reductions in the GFP intensity (Fig. 7D). Taken together, these results suggest that compound 0179841 can target the F protein for degradation and confer antiviral activity.

### Compound 0179841 inhibits RSV infection in human respiratory organoids

**Fig 8:**
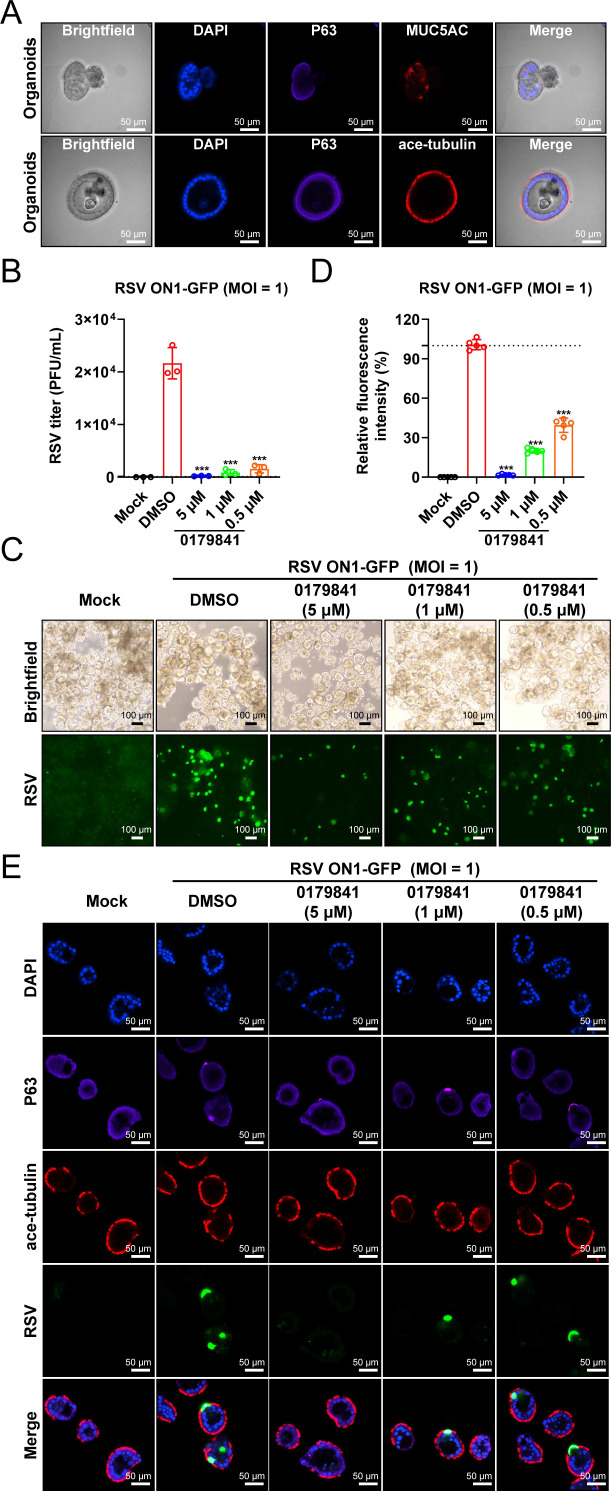
Evaluation of the antiviral activity of compound 0179841 in a human airway organoid model. (**A**) Establishment of HBEC-derived apical-out airway organoids. Representative white-light images of organoids cultured over the long term are presented, along with confocal images demonstrating the expression of respiratory epithelial markers. The confocal images illustrate the presence and spatial distribution of distinct cell types within the organoids, as identified by their specific markers: basal cells labeled with P63 (purple), goblet cells labeled with MUC5AC (green), and ciliated cells labeled with acetylated tubulin (red). (**B–E**) Organoids were infected with RSV ON1-GFP at an MOI of 1 and cultured in media supplemented with 0.1% DMSO or compound 0179841 at the indicated concentrations (5, 1, and 0.5 µM) for 96 h. (**B**) The cell culture supernatant of infected organoids was collected, and a TCID_50_ assay was performed. (**C**) The RSV ON1-GFP-infected organoids were visualized via microscopy. (**D**) The GFP intensity in five randomly selected fields per treatment was measured using ImageJ analysis software and normalized to the intensity in the DMSO condition. (**E**) The RSV ON1-GFP-infected organoids were visualized using a confocal microscope. The graphs shown are representative of three independent experiments. The differences between the experimental and DMSO groups were determined by Student’s *t*-test for comparing two groups of data (***P < 0.001).

Human respiratory organoids are widely utilized as model systems for investigating human respiratory viral pathogenesis and supporting pharmaceutical research (53–57). Here, we differentiated HBECs into mature, physiologically representative apical-out airway organoids. These organoids contain basal, secretory (goblet), and outward-facing ciliated cells, mimicking the apical surface of the airway epithelium *in vivo* (Fig. 8A). Next, we evaluated the efficacy of compound 0179841 against RSV in the human airway organoid model. Compound 0179841 significantly reduced viral loads and protein expression in a dose-dependent manner in lung airway organoids (Fig. 8B through E). Compared with the DMSO control, compound 0179841 at a concentration of 5 µM reduced viral titers by more than 100-fold (Fig. 8B). Additionally, the GFP intensity demonstrated a pronounced antiviral effect of compound 0179841 against RSV ON1-GFP (Fig. 8C through E). These data demonstrate that compound 0179841 can inhibit RSV infection in a physiologically relevant organoid system.

## DISCUSSION

Fusion inhibitor-based interventions for RSV are facing increasing resistance development *in vitro* and *in vivo*, underscoring the need for new antivirals or alternative modes of action. Lonafarnib, a novel antiviral that targets the F protein, has shown efficacy in a BALB/c mouse infection model (36, 37). Given the adaptations that RSV has already exhibited to other fusion inhibitors, understanding potential lonafarnib resistance mechanisms is crucial. The results presented herein demonstrate that multiple different mutational residues in the fusion protein of diverse RSV strains are associated with lonafarnib resistance, with a notable preference for mutations in the cysteine-rich region. Mechanistic studies indicate that resistance mutations occupy internal sites within the prefusion RSV F protein, likely destabilizing it and reducing its binding affinity for lonafarnib. Importantly, we designed and identified a lonafarnib-based antiviral degrader, compound 0179841, which inhibits RSV infection by inducing F protein degradation.

The T335I and T400A mutations in the F protein of the RSV Long strain (rHRSV-A-GFP), which were previously linked to lonafarnib resistance, were confirmed (36). Here, large-scale passaging of RSV with lonafarnib selected for resistance mutations identified K399N in the RSV A2 strain and K394R in the clinically dominant RSV ON1 strain as prevalent substitutions (>5% mutation frequency, emerging in a stepwise manner). The differing mutations observed among these strains emphasize the complexity and variety of pathways leading to lonafarnib resistance, although it is not yet clear why certain mutations are specific to a particular strain. Although the mutation sites may vary among RSV strains, all the resulting amino acid substitutions are confined to the relatively conserved cysteine-rich region of the F protein. Cell‒cell fusion analysis of these mutants and other reported fusion inhibitors confirmed resistance-associated substitutions (D392G (49), S398L (50, 58), K399I (51), T400I (52), and D401E (33)) in the region in which these amino acid mutations in the cysteine-rich region mediate high-level resistance to lonafarnib, with the exception of D392G/N (Fig. 3), which was further validated via several recombinant live RSV strains carrying single F gene mutations (Table 1). Interestingly, the D392N and K399N mutations in the RSV A2 F protein were significantly negatively correlated, suggesting that they are not independent (Fig. 1E). These findings seem to indicate that lonafarnib could induce the D392N mutation to counteract mutations at other sites in the cysteine-rich region.

Furthermore, lonafarnib resistance mutations (K394R, K399N, and T400A) confer cross-resistance to other clinically relevant fusion inhibitors (e.g., GS-5806 (14), AK0529 (15), RV521 (12), and JNJ-53718678 (13)) (Table 2). GS-5806 (14), AK0529 (24), RV521 (26), and JNJ-53718678 (59) bind to a hydrophobic pocket in the F protein that is rich in phenylalanine residues (Phe488, Phe137, and Phe140). Our recently resolved cryo-EM structure of the F protein–lonafarnib complex revealed that lonafarnib occupies the same hydrophobic cavity and β-sheet network as these inhibitors do and additionally forms hydrogen bonds with phenylalanine residues (37). This binding pocket is proximal to residues K394, K399, and T400. Collectively, these structural insights explain why the K394R, K399N, and T400A mutations confer cross-resistance to multiple fusion inhibitors, including lonafarnib. Consequently, developing effective RSV F protein inhibitors or new therapeutic options against these mutants is urgently needed before the global spread of mutant strains.

Resistance mutations to RSV fusion inhibitors are predominantly located in a central cavity adjacent to the fusion peptide within the prefusion F trimer (32, 33). These regions include (i) the fusion peptide (amino acid residues 140–144), (ii) the cysteine-rich region (residues 392–401), and (iii) the heptad repeat B (residues 486–489). Resistance can arise through direct or indirect mechanisms. Direct mechanisms include mutations at the residues that interact directly with the inhibitors (e.g., D486N and F488L) or hindrance of the conformational changes required for inhibitor binding (e.g., D489Y) (32). Indirect mechanisms alter F protein stability and triggering rates (e.g., K394R, T400A, D401E, and D489E), narrowing the window for effective inhibitor binding (33). Mutations associated with lonafarnib-resistant RSV infection are located mainly in the cysteine-rich region of the F protein (Fig. 1 to 3; Table 1). These resistance-associated substitutions result in a low binding affinity by interfering with the binding of the drug to the hydrophobic pocket (Fig. 4) but do not participate directly in the binding of lonafarnib to the F protein (37). Importantly, the SPR experiments utilized a stabilized prefusion F protein; consequently, mutations that exert allosteric effects may not be detectable in this system. All-atom MD simulations revealed that mutations affect protein stability (Fig. 5). The lower Rg and SASA values in DS-Cav1 and D392N indicate greater stability. In contrast, other mutations, such as K394R, presented the highest SASA and Rg values, indicating decreased stability. This destabilization appears to disrupt the hydrophobic cavity, and the β-sheet network is critical for inhibitor binding, even for compounds that target nonoverlapping sites. Notably, mutations such as K394R in the cysteine-rich region (aa 392–401) induce conformational changes through interconnected structural motifs (e.g., β-sheets at residues 331–335 and 486–488), thereby indirectly altering the fusion peptide and HRB regions. It is worth noting that the K394R mutant confers resistance to lonafarnib by destabilizing the F protein and increasing its membrane fusion activity (Fig. 3 and 5), along with cross-resistance to other fusion inhibitors (Table 2) (60). Therefore, it is conceivable that such an RSV mutant carrying the K394R mutation in the F protein may pose a potential future threat to lonafarnib-based therapeutics. This concern necessitates the development of next-generation inhibitors, or their combination with other agents, to treat RSV infection more effectively and prevent the emergence of drug-resistant RSV.

Since the emergence of PROTAC technology in 2001 (61), it has revolutionized drug discovery by enabling the degradation of disease-causing proteins. Over the past two decades, cancer research has undergone significant breakthroughs through the use of PROTACs to target “undruggable” oncoproteins (62). More recently, this approach has been extended to antiviral research, in which unique protein degradation pathways have been utilized to combat viral infections (46, 63). Conventional small-molecule inhibitors targeting the RSV F protein carry inherent risks of developing resistance, as demonstrated by the rapid emergence of escape mutations under selective pressure (14, 15, 21–26). To preemptively address this limitation, we pursued a degradation-based strategy using PROTACs. This approach aims to eliminate the target protein rather than modulate its activity, thereby reducing the opportunities for resistance mutations to confer survival advantages. In this study, we explored PROTAC modalities that target the RSV F protein for degradation to achieve virus-specific antiviral effects. The identification of the small-molecule degrader 0179841 demonstrates the potential for developing and rationally designing antivirals via the PROTAC platform. Compound 0179841 is a heterobifunctional small molecule composed of two ligands connected by a linker: lonafarnib (which recruits the F protein) and cereblon (which recruits E3 ubiquitin ligase) (Fig. 6). Simultaneous binding of both the F protein and the E3 ligase by compound 0179841 induces ubiquitylation of the F protein, leading to its subsequent degradation via the ubiquitin-proteasome system. Although compound 0179841 exhibited modest antiviral effects against RSV replication *in vitro* (Fig. 7 and 8), this proof-of-concept research is encouraging. Future studies will focus on optimizing PROTAC degrader sequences through structure‒activity relationship (SAR) analysis to enhance anti-RSV efficacy, pharmacokinetic properties, and immunogenicity profiles. These attributes are dictated by sequence architecture, length, and backbone configuration, which critically influence ternary complex formation and target engagement. Additionally, given that lonafarnib binds to the monomeric F protein and trimeric prefusion F protein (37), compound 0179841 may also bind inside the central cavity of the prefusion F protein to block its conformational change in addition to the degradation of the monomeric F protein. Although the dependence of compound 0179841 on lonafarnib binding means that it cannot directly overcome lonafarnib-associated resistance, as resistant mutations reduce the lonafarnib-F protein interaction (Fig. 4), it operates via a distinct antiviral mechanism—targeted degradation. Therefore, the resistance profile of 0179841 against RSV may plausibly differ from that of lonafarnib. Such insights would be invaluable for the development of novel antiviral therapeutics targeting this specific interaction, potentially offering a viable strategy against RSV.

### Limitations of the study

Sequencing of escape populations revealed L119I and K394R mutations in the F gene of RSV ON1 in response to lonafarnib, and a correlation analysis of the two mutation frequencies revealed a significant positive correlation. However, the L119 residue is located in the p27 peptide, which is removed by furin during the post-translational processing of the F protein (47, 48); thus, we were unable to verify its role in drug resistance at the protein level. Additionally, our data suggest that at the doses and within the short time frames we utilized, the replication of RSV is sensitive to compound 0179841-mediated F protein degradation *in vitro*. However, *in vivo* validation in mice is limited for the following reasons: CRBN exhibits notable species-specific differences between mice and humans, especially in terms of substrate recognition and binding properties. A humanized CRBN (hCRBN) mouse model is therefore needed. Moreover, as a virus-directed strategy targeting proteostasis, further improvement of the EC_50_ against RSV *in vitro*, as well as analyses of the long-term pharmacokinetics, toxicity profiles, and delivery efficiency to lung tissue of compound 0179841 in an *in vivo* context, is still necessary.

## MATERIALS AND METHODS

### Cells, viruses, and compounds

HEp-2 cells (cat#CCL-23, RRID: CVCL_1906) and HEK293T cells (cat#CRL-3216, RRID: CVCL_0063) were purchased from the American Type Culture Collection (ATCC) and cultured in DMEM medium (HyClone, cat#SH30022.01) supplemented with 10% FBS (Thermo Fisher Scientific, cat#10099141), 2% HEPES (Thermo Fisher Scientific, cat#15630080), and 1% penicillin-streptomycin (Life Technologies, cat#15140122) at 37°C and 5% CO_2_ in a humidified incubator. HEK293F cell lines (RRID: CVCL_6642) were provided by Professor Xiaoli Xiong from Guangzhou Institutes of Biomedicine and Health and were propagated in serum-free SMM 293-TII Expression Medium (Sino Biological Inc., cat#M293TII) using orbital shakers (160 rpm) under equivalent temperature and CO₂ conditions. Human bronchial epithelial cells (HBECs, cat#CP-H009) were isolated from bronchial tissue, purchased from Procell, and subsequently cultured in specialized medium (Procell, cat#CM-H009). All cell lines were confirmed mycoplasma-free through monthly PCR testing.

RSV A2 and RSV ON1-GFP were described elsewhere (37). Recombinant live RSV carrying V127G, L141F, K399N, T400A, T400I, or F488L mutant of F protein was a kind gift from Gang Zou (Shanghai Ark Biopharmaceutical Co., Ltd, China). Recombinant live RSV carrying the K394R mutant of the F protein was a kind gift from Wencai Ye (Jinan University, China) (60, 64). All experiments involving live RSV were conducted in the biosafety level 2 (BSL-2) facilities in Guangzhou National Laboratory (GZNL). All viruses were propagated in HEp-2 cells and subsequently subjected to viral titer determination using a fluorescence focus assay (FFA), as previously described (37). Briefly, viral titrations were performed with 10-fold serial dilutions in HEp-2 cells. Twenty-four hours after inoculation, the cell supernatants were discarded, and the cells were fixed in 4% paraformaldehyde (PFA) for 15 min and then permeabilized with 0.3% Triton X-100 for 15 min at room temperature (RT). RSV was probed using anti-RSV-FITC (GeneTex, cat#GTX36375) diluted 1:100, over a 1 h incubation at RT. Fluorescent images were acquired with an Operetta CLS High-Content Screening System (PerkinElmer). Fluorescence dots were quantified and normalized to the total nuclear count using ImageJ analysis software (RRID: SCR_003070; National Institutes of Health, NIH).

Lonafarnib (cat#HY-15136), GS-5806 (cat#HY-16727), AK0529 (cat#HY-109142), RV521 (cat#HY-123475), and JNJ-53718678 (cat#HY-112180) were purchased from MedChemExpress. Compound 0179841 was synthesized as described below.

### *In vitro* selection for RSV resistance to lonafarnib

To select for the development of drug resistance against lonafarnib, RSV A2 and RSV ON1-GFP strains were cultured in the presence of increasing concentrations of lonafarnib and passaged 18 times, respectively. Virus isolates recovered from culture at various passages were then characterized for their resistance to lonafarnib. To initiate passaging, HEp-2 cells were seeded in a 12-well plate at a density of 2  ×  10^5^ cells per well, and both lonafarnib and virus were then added the following day. Lonafarnib was initially added at 250 nM, and the virus was added at an MOI of 0.1 per well. Two days post-infection, each well was scored for cytopathic effects (CPE) on an Operetta CLS High-Content Screening System (PerkinElmer), and 500  µL of the supernatant from the well with ~30% CPE was passaged to each well in the next culture plate. The remaining supernatant and cells were stored at −80°C until further analysis. The passage culture was set up in triplicate, and passaging was performed independently. Along with the cultures passaged with lonafarnib, RSV A2 and RSV ON1-GFP strains were passaged with 0.1% DMSO in three independent wells to serve as a passage control, respectively.

### RSV full-genome sequence

The total RNA was extracted from the passaged supernatant and cell stocks using TRIzol LS reagent (Invitrogen, cat#10296028) according to the manufacturer’s instructions. Approximately 10 µg of the extracted RNA was then dispatched to Guangdong Magigen Biotechnology Co., Ltd for full-genome sequencing using the Novaseq technique. Ribosomal RNA was depleted from samples using the Ribo-off rRNA Depletion Kit (Vazyme Biotech Co., Ltd., cat#N406-01/02). Sequencing libraries were prepared with the ALFA-SEQ RNA Library Prep Kit II (Findrop Biosafety Technology [Guangzhou] Co., Ltd., cat#NRI002E) according to the manufacturer’s instructions. Constructed amplicon libraries are subjected to PE150 sequencing on the Illumina platform.

A workflow diagram of RSV single-nucleotide variant (SNV) calling is shown in Fig. S8. Briefly, raw sequencing data were processed using Fastp v0.23.2 to remove adapter sequences and low-quality reads. Reads with a quality score below 30 (Q30) and shorter than 36 bp were filtered out to ensure high-quality input for subsequent analyses (65). Filtered reads were aligned to the viral reference genome using BWA-MEM v0.7.17. The reference genome used was the RSV strain A2 or ON1, complete genome, available from the NCBI GenBank database (accession no. KT992094 or MW582528). PCR duplicates were marked and removed using Picard Tools v2.26.9. Sequencing depth was calculated using SAMtools v1.19.2 (66–68). To ensure comprehensive variant detection, we employed a multi-caller approach using five different variant calling algorithms: BCFtools v1.19 (Using htslib 1.19.1) (69), FreeBayes v1.3.6 (70), Mutect2 (GATK v4.2.5.0) (71), Strelka v2.9.10 (72), and VarScan v2.4.6 (73). Additionally, we utilized DeepVariant v1.6.0 (74), a deep learning-based variant caller, to complement our analysis. Amino acid changes resulting from the detected variants were annotated using SnpEff v5.2 (75). A custom annotation database was built using the RSV strain A2 or ON1 from the NCBI GenBank database (accession no. KT992094 or MW582528) to ensure accurate functional predictions. Frequency thresholds for reporting mutations were set at 5% for Illumina sequencing.

### Evaluation of the *in vitro* antiviral activity

The inhibitory effects of compounds on wild-type RSV, passaged viruses, and recombinant RSV were evaluated in HEp-2 cells. The cells were seeded into 96-well plates at a density of 1.3  ×  10^4^ cells per well for 20 h. The virus was then inoculated at a dose of 0.2 MOI per well, and a 3-fold dilution series of the indicated compounds was added in triplicate. Three days post-infection, the level of viral replication was quantitatively assessed by FFA, and EC_50_ values were derived by fitting a nonlinear regression curve to the data in GraphPad Prism 8.0 software (RRID: SCR_002798, Graphpad Software Inc.). EC_90_ values of lonafarnib for recombinant RSV were calculated by the formula (EC_90_ = 9^1/HillSlope*EC_50_).

The inhibitory effects of compounds on RSV ON1-GFP were evaluated in HBEC. Specifically, 1.3 × 10^4^ cells per well were seeded into 96-well plates, infected with RSV ON1-GFP at an MOI of 2, and subsequently incubated with either lonafarnib or compound 0179841. At 72 h post-infection, the infected cells were visualized using an EVOS M5000 Cell Imaging System (Thermo Fisher Scientific). The GFP intensity was quantified in five randomly selected fields per experiment using ImageJ analysis software (RRID: SCR_003070; National Institutes of Health, NIH).

### Cytotoxicity assay

HEp-2 cells (1.3 × 10^4^) were seeded in 96-well plates for 20 h. The indicated compounds in a 3-fold dilution series were added to the cells, and three wells were performed in parallel. After 72 h, the cells were incubated with DMEM containing 10% FBS and CCK8 reagent (Beyotime Biotechnology, cat#C0038) for 30 min at 37°C. The absorbance at 450 nm and 600 nm was read using the PerkinElmer EnSight reader.

### Cell–cell fusion assay

Cell–cell fusion assays were performed using a split-GFP system (GFP1-10 and GFP11) (76). Briefly, HEK293T cells stably expressing GFP1-10 were transfected with 1,000 ng of plasmids encoding either wild-type F or F mutants containing various amino acid substitutions. Alternatively, the cells were infected with RSV and treated with 0.1% DMSO or the indicated compounds at concentrations of 5, 1, and 0.5 µM. Then, the transfected or infected cells were co-cultured with HEK293T cells stably expressing GFP11 at a 1:1 ratio (8 ×  10^4^ cells/well) for 24 h in a 96-well plate. Images were acquired per well on an Operetta CLS High-Content Screening System (PerkinElmer). The GFP amounts were quantified using ImageJ analysis software (RRID: SCR_003070; National Institutes of Health, NIH).

### Protein production

The cDNAs encoding prefusion-stabilized RSV F variant DS-Cav1 were cloned into the pcDNA3.1 expression vector as previously described (19, 37). The DS-Cav1 mutants were constructed using a site-directed mutagenesis kit. The DS-Cav1 and its mutants were expressed in HEK293F cells with polyethylenimine (PEI, Polysciences Inc., cat#24765) as previously described (37). Briefly, the cell supernatant was harvested, centrifuged, and filtered at 4 days post-transfection. The complex was initially purified with Ni^2+^-NTA resin (Cytiva, cat#17531801) using an elution buffer (1 × PBS pH 7.4, 500 mM imidazole). The proteins were further purified using a Superose 6 10/300 Gl gel filtration column (Cytiva, cat#17517201) with running buffer consisting of 1 × PBS pH 7.4, then concentrated to about 1 mg/mL.

### SPR analysis

SPR assays were performed as previously described (37). Briefly, the interaction between RSV F proteins and lonafarnib was detected using a Biacore 8K (GE Healthcare) at 25°C in a multi-cycle mode. RSV F proteins were immobilized on a Series S Sensor chip CM7 (Cytiva, cat#29147020). Lonafarnib with concentrations of 0.78125, 1.5625, 3.125, 6.25, and 12.5 µM was prepared in buffer containing 10 mM PBS pH 7.4, 0.05% Tween 20, and 5% DMSO when testing interactions with RSV F proteins. The equilibrium dissociation constants (*K*_D_) for each pair of interactions were calculated using the Biacore 8K evaluation software (Cytiva).

### Molecular dynamics simulation

The molecular dynamics simulations were performed on a SingleParticle S23TL24 workstation configured with 2× Intel Xeon Gold Platinum 8352 v Processors, 512 GB of RAM, and 4× Nvidia RTX 4090. System preparation was conducted using the Protein Preparation Wizard (RRID: SCR_016749) in Schrödinger Maestro (Schrödinger Release 2020-1, Schrödinger, LLC). The simulation systems were solvated using the SPC water model and neutralized with a 150 mM NaCl buffer. Each system, containing trimeric RSV F proteins and lonafarnib, comprised approximately 125,000 atoms within an orthorhombic 10 × 10 × 10  Å^3^ cubic box. Production simulations were carried out in the NPT ensemble, maintaining a temperature of 300 K and a pressure of 1.01325 bar. The simulations, executed with Desmond (RRID: SCR_014575) using the OPLS4 force field, ran for 500 ns each, with data recorded every 500 ps. The structural representations shown in the figures were rendered using PyMOL v.3.0.4 (RRID: SCR_000305; Schrödinger, LLC).

### Molecular docking

Protein structures of RSV F (PDB: 8KG5) and CRBD (PDB: 6BN7) were prepared for docking by retaining structural monomers, correcting structures, adding hydrogen atoms, and optimizing side chains. The resulting protein complex models were imported into MOE v2022.02 (RRID: SCR_014882) software via HDOCK v1.1 (RRID: SCR_024799). The MOE Dock module was used to dock compound 0179841 with the protein complex cereblon and RSV F. The best binding poses were identified based on geometric and energy matching, yielding a binding score of −11.38. A more negative score indicates stronger binding affinity, and the selected pose was used for interaction analysis.

### Synthesis of compounds

#### tert-butyl

##### 3-(2-(2-(2-((2-(2,6-dioxopiperidin-3-yl)-1,3-dioxoisoindolin-4-yl)amino)ethoxy)ethoxy)ethoxy)propanoate (2)

In total, 2-(2,6-Dioxopiperidin-3-yl)−4-fluoroisoindoline-1,3-dione (550 mg, 1.98 mmol) and DIPEA (1.1 mL, 6.54 mmol) were added to a solution of compound 1 (500 mg, 1.8 mmol) in DMSO (8 mL). The mixture was stirred at 90°C for 2.5 h. After being cooled to RT, the mixture was diluted with H_2_O and extracted with ethyl acetate (EA), the organic layer was washed with brine and dried over Na_2_SO_4_. The solvent was evaporated under reduced pressure, then purified by flash column, eluting with a gradient of 0%–2.5% MeOH in Dichloromethane (DCM) to give compound two as an emerald green oil (180 mg, yield 19%). HRMS (ESI) *m/z*: calculated for C_26_H_35_N_3_O_9_ [M + H]^+^ 534.2446, found 534.2442.

##### 3-(2-(2-(2-((2-(2,6-dioxopiperidin-3-yl)-1,3-dioxoisoindolin-4-yl)amino)ethoxy)ethoxy)ethoxy)propanoic acid (3)

TFA (3 mL, 41 mmol) at 0°C was added to a solution of compound 2 (180 mg, 0.35 mmol) in DCM (5 mL). After the addition, the resulting mixture was stirred for 2 h while allowing the temperature to rise slowly to RT. The mixture was spun dry to remove TFA and then purified by flash column, eluting with a gradient of 0%–2.5% MeOH in DCM to give compound three as a light-yellow oil (70 mg, yield 42%), which was used for the next step directly. HRMS (ESI) *m/z*: calculated for C_22_H_27_N_3_O_9_ [M + H]^+^ 478.1820, found 478.1811.

##### 4-((2-(2-(2-(3-(4-(2-(4-((*R*)−3,10-dibromo-8-chloro-6,11-dihydro-5*H-*benzo[5,6]cyclohepta[1,2-b]pyridin-11-yl)piperidin-1-yl)−2-oxoethyl)piperidin-1-yl)−3-oxopropoxy)ethoxy)ethoxy)ethyl)amino)−2-(2,6-dioxopiperidin-3-yl)isoindoline-1,3-dione (0179841)

To a solution of compound 3 (70 mg, 0.151 mmol) in DMF (3 mL) was added (*R*)−1-(4-(3,10-dibromo-8-chloro-6,11-dihydro-5*H-*benzo[5,6]cyclohepta[1,2-b]pyridin-11-yl)piperidin-1-yl)−2-(piperidin-4-yl)ethan-1-one (85 mg, 0.138 mmol), then added HATU (83 mg, 0.302 mmol), DIPEA (95 µL, 0.453 mmol). The mixture was stirred for 3 h, then diluted with H_2_O and extracted with EA (20 mL), the organic layer was washed with brine and dried over Na_2_SO_4_. The solvent was evaporated under reduced pressure. The residue was purified by flash column chromatography, eluting with a gradient of 0%–3.3% MeOH in DCM to give compound 0179841 as a light yellow solid (70 mg, yield 46%). HPLC: 98% (t_R_ = 14.319 min); ^1^H NMR (300 MHz, CDCl_3_) *δ* = 8.86 (s, 1H), 8.45 (s, 1H), 7.56 (s, 1H), 7.54–7.43 (m, 2H), 7.17–7.05 (m, 2H), 6.91 (d, *J* = 8.4 Hz, 1H), 6.50 (s, 1H), 4.91 (d, *J* = 10.1 Hz, 2H), 4.59 (d, *J* = 12.7 Hz, 2H), 3.89–3.76 (m, 4H), 3.76–3.55 (m, 12H), 3.46 (s, 2H), 3.27 (d, *J* = 17.8 Hz, 1H), 3.01 (s, 2H), 2.93–2.70 (m, 6H), 2.61 (q, *J* = 10.5 Hz, 3H), 2.38 (s, 2H), 2.15 (d, *J* = 28.4 Hz, 4H), 1.83 (d, *J* = 12.9 Hz, 1H), 1.73 (d, *J* = 13.2 Hz, 1H), 1.38 (d, *J* = 16.7 Hz, 2H), and 1.12 (s, 2H); HRMS (ESI) *m/z*: calculated for C_48_H_55_Br_2_ClN_6_O_9_ [M + H]^+^ 1053.2159, found 1053.2100.

### Western blot

HEK293T cells were seeded in 12-well plates (80% confluence) 1 day before F plasmids transfection and compound treatment. On the day of transfection, the cell culture medium was first changed, and then, the cells were transfected with 2 µg of F plasmids. In the assays for validating the degradation of F, 10 mM DMSO stocks of the indicated compounds were added into the medium (working concentrations = 1 µM, 0.1% DMSO) for the indicated periods of time post-transfection. Cells were scraped, washed twice with PBS, and lysed with cell lysis buffer (Beyotime Biotechnology, cat#P0013), supplemented with protease inhibitor PMSF (Beyotime Biotechnology, cat#ST505) for 30 min on ice. Cell lysates were clarified by centrifugation at 10,000 × *g* for 10 min at 4°C. Protein concentrations were determined using Enhanced BCA Protein Assay Kit (Beyotime Biotechnology, cat#P0009). 5 × SDS PAGE Sample Loading Buffer (Beyotime Biotechnology, cat#P0015L) was added to the samples and boiled at 100°C for 10 min. The equal concentrations of protein samples were separated by SDS-PAGE, and proteins were transferred from gels to PVDF membranes (Merck, cat#ISEQ00010). The membranes were incubated in 5% wt/vol milk in 0.1% Tween-20/PBS at RT for 1 h, incubated with the indicated primary antibodies (mouse anti-RSV F antibody, Abcam, cat#ab43812, RRID: AB_777676; mouse anti-β-actin antibody, Proteintech, cat#66009-1-Ig, RRID: AB_2687938) diluted in 0.1% Tween-20/PBS at 4°C overnight and incubated with the indicated secondary antibodies (Peroxidase AffiniPure Goat Anti-Mouse IgG, Jackson Immuno Research, cat#115-035-146, RRID:AB_2307392) diluted in 0.1% Tween-20/PBS at RT for 1 h. The membranes were washed by 0.1% Tween-20/PBS after each incubation. Signal bands were exposed and quantified using the FluorChem HD2 system (Alpha Innotech).

### Antiviral drug assay using lung airway organoids

HBECs-derived airway organoids were kindly provided by Ning Ma (Guangzhou National Laboratory, China). In brief, HBECs were initially cultured in PneumaCult-Ex Plus Medium (STEMCELL Technologies, cat#05040), then seeded at 2 × 10⁵ cells/well into anti-adherence rinsing solution (STEMCELL Technologies, cat#07010)-treated AggreWell400 24-well plates (STEMCELL Technologies, cat#34415). They were maintained in PneumaCult Apical-Out Airway Organoid Medium (STEMCELL Technologies, cat#100-0620) for 6 days. Subsequently, the cells were transferred to similarly treated flat-bottom 24-well plates and cultured in the same medium for an additional 9 days.

The airway organoids were infected with RSV ON1-GFP at an MOI of 1 for 4 h. After removal of the inoculum, the organoids were washed twice with PBS, and the infected organoids were cultured in medium containing 0.1% DMSO or serially diluted compound 0179841. At 96 h post-infection, cell-free medium was harvested, and a TCID_50_ assay was performed to determine the viral titer. The organoids were fixed in parallel in 4% PFA, permeabilized with 0.5% Triton X-100 (1 h), blocked with 5% BSA (Macklin, cat#B824162; 2 h), and incubated overnight at 4°C with primary antibodies: Goat anti-Human p63/TP73L polyclonal antibody (R&D Systems, cat#AF1916), Rabbit anti-Human Mucin 5AC polyclonal antibody (Abways, cat#CY6826), and Mouse anti-acetylated tubulin monoclonal antibody (Sigma-Aldrich, cat#T7451). After being washed three times with PBS, the samples were stained with Donkey anti-Goat IgG (H + L) Cross-Adsorbed Secondary Antibody, Alexa Fluor 633 (Thermo Fisher Scientific, cat#A-21082; 1 hour, RT), Donkey anti-Rabbit IgG (H + L) Highly Cross-Adsorbed Secondary Antibody, Alexa Fluor 568 (Thermo Fisher Scientific, cat#A-10042; 1 h, RT), and Donkey anti-Mouse IgG (H + L) Highly Cross-Adsorbed Secondary Antibody, Alexa Fluor 568 (Thermo Fisher Scientific, cat#A-10037; 1 h, RT), followed by Hoechst 33342 nuclear counterstain (Thermo Fisher Scientific, cat#62249). After being washed three times with PBS, images were acquired on a Nikon A1 confocal microscope (Nikon). The GFP fluorescence intensity was quantified using ImageJ analysis software (RRID: SCR_003070; National Institutes of Health, NIH).

### Statistical analysis

The data in the figures represent means ± SDs and were analyzed using GraphPad Prism 8.0 software (RRID: SCR_002798, Graphpad Software Inc.). The linkage relationship between mutations was analyzed using a χ^2^ test for independence. For the cell–cell fusion assay, statistical comparisons between different groups were performed using the non-parametric Mann-Whitney test, combining data from several experiments. The differences between the experimental and control groups were determined by Student’s *t*-test involving two groups or one-way ANOVA, followed by Dunnett’s post hoc test for multiple group comparisons. *P*‐values were calculated, and statistical significance was expressed as *P < 0.05, **P < 0.01, ***P < 0.001; ns, not significant. All experiments were performed with at least three biological replicates. The details of the statistical analyses were described in the figure legends.

## Data Availability

All experimental data are provided in the article. The sequences of RSV A2 (accession no. KT992094) and RSV ON1 (accession no. MW582528) used for alignment were downloaded from GenBank. The raw NGS data from HEp-2 cells are available from the NCBI Sequence Read Archive under BioProject Accession IDs PRJNA1182833 and PRJNA1186173. The materials used in this study will be made available under an appropriate material transfer agreement. The data preprocessing and analysis pipeline is available at https://github.com/dailypartita/RSV_SNV_Calling_pipeline. It includes custom scripts for quality control, alignment, variant calling, and annotation. The pipeline utilizes the following open-source software: Fastp v0.23.2 (RRID: SCR_016962), BWA-MEM2 v0.7.17 (RRID: SCR_022192), Picard Tools v2.26.9 (RRID: SCR_006525), SAMtools v1.19.2 (RRID: SCR_002105), BCFtools v1.19 (RRID: SCR_005227), FreeBayes v1.3.6 (RRID: SCR_010761), GATK v4.2.5.0 (RRID: SCR_001876), Strelka v2.9.10 (RRID: SCR_005109), VarScan v2.4.6 (RRID: SCR_006849), DeepVariant v1.6.0 (Google Brain, Verily Life Sciences), and SnpEff v5.2 (RRID: SCR_005191). The RSV strains A2 (accession no. KT992094) and ON1 (accession no. MW582528) were used for alignment and variant calling.

## SUPPLEMENTAL MATERIAL

Supplemental material jvi.01487-25-s0001.docx Tables S1 and S2; Fig. S1 to S8.

ASM does not own the copyrights to Supplemental Material that may be linked to, or accessed through, an article. The authors have granted ASM a non-exclusive, world-wide license to publish the Supplemental Material files. Please contact the corresponding author directly for reuse.

## References

[1] Li Y, Wang X, Blau DM, Caballero MT, Feikin DR, Gill CJ, Madhi SA, Omer SB, Simões EAF, Campbell H, et al.. 2022. Global, regional, and national disease burden estimates of acute lower respiratory infections due to respiratory syncytial virus in children younger than 5 years in 2019: a systematic analysis. The Lancet 399:2047–2064. doi:10.1016/S0140-6736(22)00478-0 PMC7613574 35598608

[2] Nguyen-Van-Tam JS, O’Leary M, Martin ET, Heijnen E, Callendret B, Fleischhackl R, Comeaux C, Tran TMP, Weber K. 2022. Burden of respiratory syncytial virus infection in older and high-risk adults: a systematic review and meta-analysis of the evidence from developed countries. Eur Respir Rev 31:220105. doi:10.1183/16000617.0105-2022 36384703 PMC9724807

[3] Papi A, Ison MG, Langley JM, Lee D-G, Leroux-Roels I, Martinon-Torres F, Schwarz TF, van Zyl-Smit RN, Campora L, Dezutter N, de Schrevel N, Fissette L, David M-P, Van der Wielen M, Kostanyan L, Hulstrøm V. 2023. Respiratory syncytial virus prefusion f protein vaccine in older adults. N Engl J Med 388:595–608. doi:10.1056/NEJMoa2209604 36791160

[4] Walsh EE, Pérez Marc G, Zareba AM, Falsey AR, Jiang Q, Patton M, Polack FP, Llapur C, Doreski PA, Ilangovan K, et al.. 2023. Efficacy and safety of a bivalent RSV prefusion F vaccine in older adults. N Engl J Med 388:1465–1477. doi:10.1056/NEJMoa2213836 37018468

[5] Wilson E, Goswami J, Baqui AH, Doreski PA, Perez-Marc G, Zaman K, Monroy J, Duncan CJA, Ujiie M, Rämet M, et al.. 2023. Efficacy and safety of an mRNA-based RSV PreF vaccine in older adults. N Engl J Med 389:2233–2244. doi:10.1056/NEJMoa2307079 38091530

[6] The IMpact-RSV Study Group. 1998. Palivizumab, a humanized respiratory syncytial virus monoclonal antibody, reduces hospitalization from respiratory syncytial virus infection in high-risk infants. Pediatrics 102:531–537. doi:10.1542/peds.102.3.531 9724660

[7] Jones JM, Fleming-Dutra KE, Prill MM, Roper LE, Brooks O, Sánchez PJ, Kotton CN, Mahon BE, Meyer S, Long SS, McMorrow ML. 2023. Use of nirsevimab for the prevention of respiratory syncytial virus disease among infants and young children: recommendations of the advisory committee on immunization practices - United States, 2023. MMWR Morb Mortal Wkly Rep 72:920–925. doi:10.15585/mmwr.mm7234a4 37616235 PMC10468217

[8] Anastassopoulou C, Medić S, Ferous S, Boufidou F, Tsakris A. 2025. Development, current status, and remaining challenges for respiratory syncytial virus vaccines. Vaccines (Basel) 13:97. doi:10.3390/vaccines13020097 40006644 PMC11860200

[9] Moderna. 2024 Review of investigational RSV (mRNA-1345) and RSV/hMPV (mRNA-1365) vaccines in infants and children < 2 Years. Available from: https://www.fda.gov/media/184378/download

[10] Cieslak CM. 2024. Nirsevimab immunization to prevent respiratory syncytial virus-associated lower respiratory tract infections in infants and children up to 24 months of age. Nurs Womens Health 28:75–79. doi:10.1016/j.nwh.2023.11.002 38070539

[11] vaxart. 2016. Safety, Efficacy and Pharmacokinetics of BTA-C585 in a RSV Viral Challenge Study (NCT02718937). Available from: https://clinicaltrials.gov/study/NCT02718937

[12] DeVincenzo J, Tait D, Efthimiou J, Mori J, Kim YI, Thomas E, Wilson L, Harland R, Mathews N, Cockerill S, Powell K, Littler E. 2020. A randomized, placebo-controlled, respiratory syncytial virus human challenge study of the antiviral efficacy, safety, and pharmacokinetics of RV521, an inhibitor of the RSV-F protein. Antimicrob Agents Chemother 64:e01884-19. doi:10.1128/AAC.01884-19 31712214 PMC6985722

[13] Martinón-Torres F, Rusch S, Huntjens D, Remmerie B, Vingerhoets J, McFadyen K, Ferrero F, Baraldi E, Rojo P, Epalza C, Stevens M. 2020. Pharmacokinetics, safety, and antiviral effects of multiple doses of the respiratory syncytial virus (RSV) fusion protein inhibitor, JNJ-53718678, in infants hospitalized with RSV infection: a randomized phase 1b study. Clin Infect Dis 71:e594–e603. doi:10.1093/cid/ciaa283 32201897 PMC7744997

[14] Porter DP, Guo Y, Perry J, Gossage DL, Watkins TR, Chien JW, Jordan R. 2020. Assessment of drug resistance during phase 2b clinical trials of presatovir in adults naturally infected with respiratory syncytial virus. Antimicrob Agents Chemother 64:e02312-19. doi:10.1128/AAC.02312-19 32071058 PMC7449164

[15] Zhao S, Shang Y, Yin Y, Zou Y, Xu Y, Zhong L, Zhang H, Zhang H, Zhao D, Shen T, et al.. 2024. Ziresovir in hospitalized infants with respiratory syncytial virus infection. N Engl J Med 391:1096–1107. doi:10.1056/NEJMoa2313551 39321361

[16] Ahmad A, Eze K, Noulin N, Horvathova V, Murray B, Baillet M, Grey L, Mori J, Adda N. 2022. EDP-938, a respiratory syncytial virus inhibitor, in a human virus challenge. N Engl J Med 386:655–666. doi:10.1056/NEJMoa2108903 35172056

[17] DeVincenzo JP, McClure MW, Symons JA, Fathi H, Westland C, Chanda S, Lambkin-Williams R, Smith P, Zhang Q, Beigelman L, Blatt LM, Fry J. 2015. Activity of oral ALS-008176 in a respiratory syncytial virus challenge study. N Engl J Med 373:2048–2058. doi:10.1056/NEJMoa1413275 26580997

[18] DeVincenzo J, Cass L, Murray A, Woodward K, Meals E, Coates M, Daly L, Wheeler V, Mori J, Brindley C, Davis A, McCurdy M, Ito K, Murray B, Strong P, Rapeport G. 2022. Safety and antiviral effects of nebulized PC786 in a respiratory syncytial virus challenge study. J Infect Dis 225:2087–2096. doi:10.1093/infdis/jiaa716 33216113 PMC9200148

[19] McLellan JS, Chen M, Joyce MG, Sastry M, Stewart-Jones GBE, Yang Y, Zhang B, Chen L, Srivatsan S, Zheng A, et al.. 2013. Structure-based design of a fusion glycoprotein vaccine for respiratory syncytial virus. Science 342:592–598. doi:10.1126/science.1243283 24179220 PMC4461862

[20] Griffiths CD, Bilawchuk LM, McDonough JE, Jamieson KC, Elawar F, Cen Y, Duan W, Lin C, Song H, Casanova JL, Ogg S, Jensen LD, Thienpont B, Kumar A, Hobman TC, Proud D, Moraes TJ, Marchant DJ. 2020. IGF1R is an entry receptor for respiratory syncytial virus. Nature 583:615–619. doi:10.1038/s41586-020-2369-7 32494007

[21] Perron M, Stray K, Kinkade A, Theodore D, Lee G, Eisenberg E, Sangi M, Gilbert BE, Jordan R, Piedra PA, Toms GL, Mackman R, Cihlar T. 2016. GS-5806 inhibits a broad range of respiratory syncytial virus clinical isolates by blocking the virus-cell fusion process. Antimicrob Agents Chemother 60:1264–1273. doi:10.1128/AAC.01497-15 PMC4776015 26666922

[22] Roymans D, Alnajjar SS, Battles MB, Sitthicharoenchai P, Furmanova-Hollenstein P, Rigaux P, Berg JV den, Kwanten L, Ginderen MV, Verheyen N, Vranckx L, Jaensch S, Arnoult E, Voorzaat R, Gallup JM, Larios-Mora A, Crabbe M, Huntjens D, Raboisson P, Langedijk JP, Ackermann MR, McLellan JS, Vendeville S, Koul A. 2017. Therapeutic efficacy of a respiratory syncytial virus fusion inhibitor. Nat Commun 8:167. doi:10.1038/s41467-017-00170-x 28761099 PMC5537225

[23] Zheng X, Liang C, Wang L, Wang B, Liu Y, Feng S, Wu JZ, Gao L, Feng L, Chen L, Guo T, Shen HC, Yun H. 2018. Discovery of benzoazepinequinoline (BAQ) derivatives as novel, potent, orally bioavailable respiratory syncytial virus fusion inhibitors. J Med Chem 61:10228–10241. doi:10.1021/acs.jmedchem.8b01394 30339388

[24] Zheng X, Gao L, Wang L, Liang C, Wang B, Liu Y, Feng S, Zhang B, Zhou M, Yu X, Xiang K, Chen L, Guo T, Shen HC, Zou G, Wu JZ, Yun H. 2019. Discovery of ziresovir as a potent, selective, and orally bioavailable respiratory syncytial virus fusion protein inhibitor. J Med Chem 62:6003–6014. doi:10.1021/acs.jmedchem.9b00654 31194544

[25] Yoshida I, Arikawa K, Honma Y, Inatani S, Yoshinaga M, Sugiyama H. 2020. Pharmacological characterization of TP0591816, a Novel macrocyclic respiratory syncytial virus fusion inhibitor with antiviral activity against F protein mutants. Antimicrob Agents Chemother 65:e01407-20. doi:10.1128/AAC.01407-20 33046486 PMC7927798

[26] Cockerill GS, Angell RM, Bedernjak A, Chuckowree I, Fraser I, Gascon-Simorte J, Gilman MSA, Good JAD, Harland R, Johnson SM, et al.. 2021. Discovery of sisunatovir (RV521), an inhibitor of respiratory syncytial virus fusion. J Med Chem 64:3658–3676. doi:10.1021/acs.jmedchem.0c01882 33729773

[27] Coates M, Brookes D, Kim YI, Allen H, Fordyce EAF, Meals EA, Colley T, Ciana CL, Parra GF, Sherbukhin V, Stockwell JA, Thomas JC, Hunt SF, Anderson-Dring L, Onions ST, Cass L, Murray PJ, Ito K, Strong P, DeVincenzo JP, Rapeport G. 2017. Preclinical characterization of PC786, an inhaled small-molecule respiratory syncytial virus L protein polymerase inhibitor. Antimicrob Agents Chemother 61:e00737-17. doi:10.1128/AAC.00737-17 28652242 PMC5571287

[28] Rhodin MHJ, McAllister NV, Castillo J, Noton SL, Fearns R, Kim IJ, Yu J, Blaisdell TP, Panarese J, Shook BC, Or YS, Goodwin B, Lin K. 2021. EDP-938, a novel nucleoprotein inhibitor of respiratory syncytial virus, demonstrates potent antiviral activities in vitro and in a non-human primate model. PLoS Pathog 17:e1009428. doi:10.1371/journal.ppat.1009428 33720995 PMC7993833

[29] Zhao X, Singh M, Malashkevich VN, Kim PS. 2000. Structural characterization of the human respiratory syncytial virus fusion protein core. Proc Natl Acad Sci USA 97:14172–14177. doi:10.1073/pnas.260499197 11106388 PMC18890

[30] Kahn JS, Schnell MJ, Buonocore L, Rose JK. 1999. Recombinant vesicular stomatitis virus expressing respiratory syncytial virus (RSV) glycoproteins: RSV fusion protein can mediate infection and cell fusion. Virology (Auckl) 254:81–91. doi:10.1006/viro.1998.9535 9927576

[31] Techaarpornkul S, Barretto N, Peeples ME. 2001. Functional analysis of recombinant respiratory syncytial virus deletion mutants lacking the small hydrophobic and/or attachment glycoprotein gene. J Virol 75:6825–6834. doi:10.1128/JVI.75.15.6825-6834.2001 11435561 PMC114409

[32] Battles MB, Langedijk JP, Furmanova-Hollenstein P, Chaiwatpongsakorn S, Costello HM, Kwanten L, Vranckx L, Vink P, Jaensch S, Jonckers THM, Koul A, Arnoult E, Peeples ME, Roymans D, McLellan JS. 2016. Molecular mechanism of respiratory syncytial virus fusion inhibitors. Nat Chem Biol 12:87–93. doi:10.1038/nchembio.1982 26641933 PMC4731865

[33] Yan D, Lee S, Thakkar VD, Luo M, Moore ML, Plemper RK. 2014. Cross-resistance mechanism of respiratory syncytial virus against structurally diverse entry inhibitors. Proc Natl Acad Sci U S A 111:E3441–E3449. doi:10.1073/pnas.1405198111 25092342 PMC4143008

[34] Young SG, Yang SH, Davies BSJ, Jung H-J, Fong LG. 2013. Targeting protein prenylation in progeria. Sci Transl Med 5:171ps3. doi:10.1126/scitranslmed.3005229 PMC3725554 23390246

[35] Urban S, Neumann-Haefelin C, Lampertico P. 2021. Hepatitis D virus in 2021: virology, immunology and new treatment approaches for a difficult-to-treat disease. Gut 70:1782–1794. doi:10.1136/gutjnl-2020-323888 34103404 PMC8355886

[36] Sake SM, Zhang X, Rajak MK, Urbanek-Quaing M, Carpentier A, Gunesch AP, Grethe C, Matthaei A, Rückert J, Galloux M, et al.. 2024. Drug repurposing screen identifies lonafarnib as respiratory syncytial virus fusion protein inhibitor. Nat Commun 15:1173. doi:10.1038/s41467-024-45241-y 38332002 PMC10853176

[37] Yang Q, Xue B, Liu F, Lu Y, Tang J, Yan M, Wu Q, Chen R, Zhou A, Liu L, Liu J, Qu C, Wu Q, Fu M, Zhong J, Dong J, Chen S, Wang F, Zhou Y, Zheng J, Peng W, Shang J, Chen X. 2024. Farnesyltransferase inhibitor lonafarnib suppresses respiratory syncytial virus infection by blocking conformational change of fusion glycoprotein. Sig Transduct Target Ther 9:144. doi:10.1038/s41392-024-01858-5 PMC11163014 38853183

[38] Ho JSY, Zhu Z, Marazzi I. 2021. Unconventional viral gene expression mechanisms as therapeutic targets. Nature 593:362–371. doi:10.1038/s41586-021-03511-5 34012080

[39] Alabi SB, Crews CM. 2021. Major advances in targeted protein degradation: PROTACs, LYTACs, and MADTACs. J Biol Chem 296:100647. doi:10.1016/j.jbc.2021.100647 33839157 PMC8131913

[40] Békés M, Langley DR, Crews CM. 2022. PROTAC targeted protein degraders: the past is prologue. Nat Rev Drug Discov 21:181–200. doi:10.1038/s41573-021-00371-6 35042991 PMC8765495

[41] Montrose K, Krissansen GW. 2014. Design of a PROTAC that antagonizes and destroys the cancer-forming X-protein of the hepatitis B virus. Biochem Biophys Res Commun 453:735–740. doi:10.1016/j.bbrc.2014.10.006 25305486

[42] de Wispelaere M, Du G, Donovan KA, Zhang T, Eleuteri NA, Yuan JC, Kalabathula J, Nowak RP, Fischer ES, Gray NS, Yang PL. 2019. Small molecule degraders of the hepatitis C virus protease reduce susceptibility to resistance mutations. Nat Commun 10:3468. doi:10.1038/s41467-019-11429-w 31371704 PMC6672008

[43] Xu Z, Liu X, Ma X, Zou W, Chen Q, Chen F, Deng X, Liang J, Dong C, Lan K, Wu S, Zhou HB. 2022. Discovery of oseltamivir-based novel PROTACs as degraders targeting neuraminidase to combat H1N1 influenza virus. Cell Insight 1:100030. doi:10.1016/j.cellin.2022.100030 37193052 PMC10120310

[44] Zhao J, Wang J, Pang X, Liu Z, Li Q, Yi D, Zhang Y, Fang X, Zhang T, Zhou R, Zhang T, Guo Z, Liu W, Li X, Liang C, Deng T, Guo F, Yu L, Cen S. 2022. An anti-influenza A virus microbial metabolite acts by degrading viral endonuclease PA. Nat Commun 13:2079. doi:10.1038/s41467-022-29690-x 35440123 PMC9019042

[45] Shaheer M, Singh R, Sobhia ME. 2022. Protein degradation: a novel computational approach to design protein degrader probes for main protease of SARS-CoV-2. J Biomol Struct Dyn 40:10905–10917. doi:10.1080/07391102.2021.1953601 34328382

[46] Zhao N, Ho JSY, Meng F, Zheng S, Kurland AP, Tian L, Rea-Moreno M, Song X, Seo J-S, Kaniskan HÜ, te Velthuis AJW, Tortorella D, Chen Y-W, Johnson JR, Jin J, Marazzi I. 2023. Generation of host-directed and virus-specific antivirals using targeted protein degradation promoted by small molecules and viral RNA mimics. Cell Host & Microbe 31:1154–1169. doi:10.1016/j.chom.2023.05.030 37339625 PMC10528416

[47] McLellan JS, Chen M, Leung S, Graepel KW, Du X, Yang Y, Zhou T, Baxa U, Yasuda E, Beaumont T, Kumar A, Modjarrad K, Zheng Z, Zhao M, Xia N, Kwong PD, Graham BS. 2013. Structure of RSV fusion glycoprotein trimer bound to a prefusion-specific neutralizing antibody. Science 340:1113–1117. doi:10.1126/science.1234914 23618766 PMC4459498

[48] Krzyzaniak MA, Zumstein MT, Gerez JA, Picotti P, Helenius A. 2013. Host cell entry of respiratory syncytial virus involves macropinocytosis followed by proteolytic activation of the F protein. PLoS Pathog 9:e1003309. doi:10.1371/journal.ppat.1003309 23593008 PMC3623752

[49] Cianci C, Yu KL, Combrink K, Sin N, Pearce B, Wang A, Civiello R, Voss S, Luo G, Kadow K, Genovesi EV, Venables B, Gulgeze H, Trehan A, James J, Lamb L, Medina I, Roach J, Yang Z, Zadjura L, Colonno R, Clark J, Meanwell N, Krystal M. 2004. Orally active fusion inhibitor of respiratory syncytial virus. Antimicrob Agents Chemother 48:413–422. doi:10.1128/AAC.48.2.413-422.2004 14742189 PMC321540

[50] Roymans D, De Bondt HL, Arnoult E, Geluykens P, Gevers T, Van Ginderen M, Verheyen N, Kim H, Willebrords R, Bonfanti J-F, Bruinzeel W, Cummings MD, van Vlijmen H, Andries K. 2010. Binding of a potent small-molecule inhibitor of six-helix bundle formation requires interactions with both heptad-repeats of the RSV fusion protein. Proc Natl Acad Sci USA 107:308–313. doi:10.1073/pnas.0910108106 19966279 PMC2806771

[51] Douglas JL, Panis ML, Ho E, Lin KY, Krawczyk SH, Grant DM, Cai R, Swaminathan S, Chen X, Cihlar T. 2005. Small molecules VP-14637 and JNJ-2408068 inhibit respiratory syncytial virus fusion by similar mechanisms. Antimicrob Agents Chemother 49:2460–2466. doi:10.1128/AAC.49.6.2460-2466.2005 15917547 PMC1140497

[52] Lundin A, Bergström T, Bendrioua L, Kann N, Adamiak B, Trybala E. 2010. Two novel fusion inhibitors of human respiratory syncytial virus. Antiviral Res 88:317–324. doi:10.1016/j.antiviral.2010.10.004 20965215

[53] Hashimoto R, Watanabe Y, Keshta A, Sugiyama M, Kitai Y, Hirabayashi A, Yasuhara N, Morimoto S, Sakamoto A, Matsumura Y, Nishimura H, Noda T, Yamamoto T, Nagao M, Takeda M, Takayama K. 2025. Human iPS cell–derived respiratory organoids as a model for respiratory syncytial virus infection. Life Sci Alliance 8:e202402837. doi:10.26508/lsa.202402837 40262853 PMC12015132

[54] Sun J, Sun D, Yang Q, Wang D, Peng J, Guo H, Ding X, Chen Z, Yuan B, Ivanenkov YA, et al.. 2025. A novel, covalent broad-spectrum inhibitor targeting human coronavirus Mpro. Nat Commun 16:4546. doi:10.1038/s41467-025-59870-4 40374668 PMC12081877

[55] Tang X, Xue D, Zhang T, Nilsson-Payant BE, Carrau L, Duan X, Gordillo M, Tan AY, Qiu Y, Xiang J, Schwartz RE, tenOever BR, Evans T, Chen S. 2023. A multi-organoid platform identifies CIART as a key factor for SARS-CoV-2 infection. Nat Cell Biol 25:381–389. doi:10.1038/s41556-023-01095-y 36918693 PMC10014579

[56] Rajan A, Weaver AM, Aloisio GM, Jelinski J, Johnson HL, Venable SF, McBride T, Aideyan L, Piedra F-A, Ye X, Melicoff-Portillo E, Yerramilli MRK, Zeng X-L, Mancini MA, Stossi F, Maresso AW, Kotkar SA, Estes MK, Blutt S, Avadhanula V, Piedra PA. 2022. The human nose organoid respiratory virus model: an ex vivo human challenge model to study respiratory syncytial virus (RSV) and severe acute respiratory syndrome coronavirus 2 (SARS-CoV-2) pathogenesis and evaluate therapeutics. mBio 13:e0351121. doi:10.1128/mbio.03511-21 PMC8844923 35164569

[57] Zhu L, Yang W, Luo J, Lu D, Hu Y, Zhang R, Li Y, Qiu L, Chen Z, Chen L, Liu H. 2025. Comparison of characteristics and immune responses between paired human nasal and bronchial epithelial organoids. Cell Biosci 15:18. doi:10.1186/s13578-024-01342-1 39920853 PMC11806626

[58] Andries K, Moeremans M, Gevers T, Willebrords R, Sommen C, Lacrampe J, Janssens F, Wyde PR. 2003. Substituted benzimidazoles with nanomolar activity against respiratory syncytial virus. Antiviral Res 60:209–219. doi:10.1016/j.antiviral.2003.07.004 14638397

[59] Cichero E, Calautti A, Francesconi V, Tonelli M, Schenone S, Fossa P. 2021. Probing in silico the benzimidazole privileged scaffold for the development of drug-like Anti-RSV agents. Pharmaceuticals (Basel) 14:1307. doi:10.3390/ph14121307 34959708 PMC8707824

[60] Tang W, Li Y, Song Q, Wang Z, Li M, Zhang Q, Wang Y, Ye W, Li Y. 2021. Mechanism of cross-resistance to fusion inhibitors conferred by the K394R mutation in respiratory syncytial virus fusion protein. J Virol 95:e0120521. doi:10.1128/JVI.01205-21 34379500 PMC8475503

[61] Sakamoto KM, Kim KB, Kumagai A, Mercurio F, Crews CM, Deshaies RJ. 2001. Protacs: chimeric molecules that target proteins to the Skp1-Cullin-F box complex for ubiquitination and degradation. Proc Natl Acad Sci USA 98:8554–8559. doi:10.1073/pnas.141230798 11438690 PMC37474

[62] Dale B, Cheng M, Park K-S, Kaniskan HÜ, Xiong Y, Jin J. 2021. Advancing targeted protein degradation for cancer therapy. Nat Rev Cancer 21:638–654. doi:10.1038/s41568-021-00365-x 34131295 PMC8463487

[63] Alugubelli YR, Xiao J, Khatua K, Kumar S, Sun L, Ma Y, Ma XR, Vulupala VR, Atla S, Blankenship LR, Coleman D, Xie X, Neuman BW, Liu WR, Xu S. 2024. Discovery of first-in-class PROTAC degraders of SARS-CoV-2 main protease. J Med Chem 67:6495–6507. doi:10.1021/acs.jmedchem.3c02416 38608245 PMC11056980

[64] Song Q, Zhu H, Qiu M, Cai J, Hu Y, Yang H, Rao S, Li Y, Li M, Hu L, Wang S, Hong J, Ye W, Chen H, Wang Y, Tang W. 2024. A new mechanism of respiratory syncytial virus entry inhibition by small-molecule to overcome K394R-associated resistance. mBio 15:e0138524. doi:10.1128/mbio.01385-24 39162560 PMC11389407

[65] Chen S, Zhou Y, Chen Y, Gu J. 2018. fastp: an ultra-fast all-in-one FASTQ preprocessor. Bioinformatics 34:i884–i890. doi:10.1093/bioinformatics/bty560 30423086 PMC6129281

[66] Li Heng, Handsaker B, Wysoker A, Fennell T, Ruan J, Homer N, Marth G, Abecasis G, Durbin R, 1000 Genome Project Data Processing Subgroup. 2009. The sequence alignment/map format and SAMtools. Bioinformatics 25:2078–2079. doi:10.1093/bioinformatics/btp352 19505943 PMC2723002

[67] Li H. 2013. Aligning sequence reads, clone sequences and assembly contigs with BWA-MEM arXiv. doi:10.48550/arXiv.1303.3997

[68] Picard toolkit. 2019. Broad Institute, GitHub Repository. https://broadinstitute.github.io/picard/.

[69] Danecek P, Bonfield JK, Liddle J, Marshall J, Ohan V, Pollard MO, Whitwham A, Keane T, McCarthy SA, Davies RM, Li H. 2021. Twelve years of SAMtools and BCFtools. Gigascience 10:giab008. doi:10.1093/gigascience/giab008 33590861 PMC7931819

[70] Garrison E, Marth G. 2012. Haplotype-based variant detection from short-read sequencing. arXiv. doi:10.48550/arXiv.1207.3907

[71] Benjamin D, Sato T, Cibulskis K, Getz G, Stewart C, Lichtenstein L. 2019. Calling somatic SNVs and Indels with Mutect2. Bioinformatics. doi:10.1101/861054

[72] Kim S, Scheffler K, Halpern AL, Bekritsky MA, Noh E, Källberg M, Chen X, Kim Y, Beyter D, Krusche P, Saunders CT. 2018. Strelka2: fast and accurate calling of germline and somatic variants. Nat Methods 15:591–594. doi:10.1038/s41592-018-0051-x 30013048

[73] Koboldt DC, Zhang Q, Larson DE, Shen D, McLellan MD, Lin L, Miller CA, Mardis ER, Ding L, Wilson RK. 2012. VarScan 2: somatic mutation and copy number alteration discovery in cancer by exome sequencing. Genome Res 22:568–576. doi:10.1101/gr.129684.111 22300766 PMC3290792

[74] Poplin R, Chang PC, Alexander D, Schwartz S, Colthurst T, Ku A, Newburger D, Dijamco J, Nguyen N, Afshar PT, Gross SS, Dorfman L, McLean CY, DePristo MA. 2018. A universal SNP and small-indel variant caller using deep neural networks. Nat Biotechnol 36:983–987. doi:10.1038/nbt.4235 30247488

[75] Cingolani P, Platts A, Wang LL, Coon M, Nguyen T, Wang L, Land SJ, Lu X, Ruden DM. 2012. A program for annotating and predicting the effects of single nucleotide polymorphisms, SnpEff: SNPs in the genome of Drosophila melanogaster strain w1118; iso-2; iso-3. Fly (Austin) 6:80–92. doi:10.4161/fly.19695 22728672 PMC3679285

[76] Cabantous S, Waldo GS. 2006. In vivo and in vitro protein solubility assays using split GFP. Nat Methods 3:845–854. doi:10.1038/nmeth932 16990817

