# Supplementary material for: Molecular mechanism of resistance to lonafarnib conferred by mutations in the cysteine-rich region of respiratory syncytial virus fusion glycoprotein and discovery of a lonafarnib-derived antiviral PROTAC
Source: J Virol. 2025 Dec 9;100(1):e01487-25. doi: 10.1128/jvi.01487-25 (PMC12817919; doi:10.1128/jvi.01487-25)
Supplement: Supplemental material — Tables S1 and S2; Fig. S1 to S8. [file jvi.01487-25-s0001.docx]

**Supplementary Information for**

**Molecular mechanism of resistance to lonafarnib conferred by mutations in the cysteine-rich region of respiratory syncytial virus fusion glycoprotein** **and discovery of a lonafarnib-derived antiviral PROTAC**

Qi Yang^1, 2, #, *^, Bao Xue^1, 3, 4, #^, Xianjie Qiu^1, #^, Kaixin Yang^1, #^, Jielin Tang^1, 2, #^, Anqi Zhou^1^, Jingjing Zou^1^, Yuhan Mao^1^, Jiayi Zhong^1^, Yuan Zhou^1^, Wei Zhang^1^, Qiong Zhang^1^, Qingyu Xiao^1^, Wei Tang^5^, Zhiyu Li^6^, Wencai Ye^5^, Gang Zou^7^, Wei Peng^1, 2, *^, Jinsai Shang^1, *^, Xi Xu^6, *^, Yixue Li^1, *^, Xinwen Chen^1, 2, *^

^1^Guangzhou National Laboratory, Guangzhou, Guangdong, 510005, P.R. China

^2^State Key Laboratory of Respiratory Disease, Guangzhou Medical University, Guangzhou, Guangdong, 511436, P.R. China

^3^Wuhan Institute of Virology, Chinese Academy of Sciences, Wuhan, 430071, P.R. China

^4^University of Chinese Academy of Sciences, Beijing 100049, P.R. China

^5^State Key Laboratory of Bioactive Molecules and Druggability Assessment, Jinan University, Guangzhou, Guangdong, 511443, P.R. China

^6^Department of Medicinal Chemistry, School of Pharmacy, China Pharmaceutical University, 24 Tongjiaxiang, Nanjing 210009, P.R. China

^7^Shanghai Ark Biopharmaceutical Co., Ltd, Shanghai, 201203, P.R. China

^#^These authors contributed equally: Qi Yang, Bao Xue, Xianjie Qiu, Kaixin Yang, Jielin Tang. Qi Yang conceived and designed the study, carried out the experiments, analyzed the data, and took the lead in writing the manuscript. Bao Xue was primarily responsible for *in vitro* passaging and antiviral assays. Xianjie Qiu conducted the molecular dynamics simulations and computational analyses. Kaixin Yang performed the RSV full-genome sequence analysis and generated the workflow diagram for RSV single nucleotide variant calling. Jielin Tang was mainly responsible for project management and contributed to manuscript writing.

^*^Correspondence: Qi Yang ([yang_qi@gzlab.ac.cn](mailto:yang_qi@gzlab.ac.cn)), Wei Peng ([peng_wei@gzlab.ac.cn](mailto:peng_wei@gzlab.ac.cn)), Jinsai Shang ([shang_jinsai@gzlab.ac.cn](mailto:peng_wei@gzlab.ac.cn)), Xi Xu (xuxi@cpu.edu.cn), Yixue Li (li_yixue@gzlab.ac.cn), Xinwen Chen (chen_xinwen@gzlab.ac.cn)

**This file includes:**

Supplementary Tables 1 and 2

Supplementary Figures 1-8

**SUPPLEMENTARY TABLES**

**TABLE S1 Mutant frequency of the fusion protein during passaging of the RSV A2 strain with lonafarnib**

| Passage | | Sequencing average depth | Control  residue  D392 | Average mutation frequency | Control  residue  K399 | Average mutation frequency |
| --- | --- | --- | --- | --- | --- | --- |
| Lineage **1** passages | P3 | 3972.56 | ● | 0% | ● | 0% |
|  | P6 | 5131.06 | ● | 0% | ● | 0% |
|  | P9 | 1721.78 | ● | 0% | N | 3.20% |
|  | P12 | 2470.83 | N | 2.00% | N | 41.15% |
|  | P15 | 1879.44 | N | 6.41% | N | 58.95% |
|  | P18 | 2270.92 | N | 7.82% | N | 55.48% |
| Lineage **2** passages | P3 | 2556.51 | ● | 0% | ● | 0% |
|  | P6 | 2884.42 | ● | 0% | ● | 0% |
|  | P9 | 2351.06 | ● | 0% | N | 2.60% |
|  | P12 | 4253.65 | N | 7.99% | N | 50.00% |
|  | P15 | 3381.56 | N | 7.53% | N | 55.43% |
|  | P18 | 1935.49 | N | 5.34% | N | 60.44% |
| Lineage **3** passages | P3 | 2317.34 | ● | 0% | ● | 0% |
|  | P6 | 2678.98 | ● | 0% | ● | 0% |
|  | P9 | 1279.20 | N | 22.11% | N | 16.48% |
|  | P12 | 1181.35 | N | 22.08% | N | 18.50% |
|  | P15 | 1231.61 | N | 24.64% | N | 16.72% |
|  | P18 | 4222.76 | N | 6.99% | N | 61.09% |

| <5% | 5-24% | 25-49% | 50-74% | 75-100% |
| --- | --- | --- | --- | --- |

**TABLE S2 Mutant frequency of the fusion protein during passaging of the RSV ON1-GFP strain with lonafarnib**

| Passage | | Sequencing average depth | T72 | Average mutation frequency | V76 | Average mutation frequency | K80 | Average mutation frequency | L119 | Average mutation frequency | T335 | Average mutation frequency | K394 | Average mutation frequency |
| --- | --- | --- | --- | --- | --- | --- | --- | --- | --- | --- | --- | --- | --- | --- |
| Lineage **1** passages | P3 | 995.73 | ● | 0% | ● | 0% | ● | 0% | ● | 0% | ● | 0% | ● | 0% |
|  | P6 | 1108.52 | ● | 0% | ● | 0% | ● | 0% | I | 2.28% | ● | 0% | R | 2.03% |
|  | P9 | 4166.55 | ● | 0% | ● | 0% | ● | 0% | I | 1.41% | ● | 0% | R | 2.12% |
|  | P12 | 1260.33 | ● | 0% | ● | 0% | ● | 0% | I | 15.21% | ● | 0% | R | 16.31% |
|  | P15 | 1077.22 | ● | 0% | A | 2.62% | N | 4.07% | I | 62.74% | ● | 0% | R | 63.77% |
|  | P18 | 4230.54 | ● | 0% | A | 7.96% | N | 7.83% | I | 59.93% | ● | 0% | R | 63.74% |
| Lineage **2** passages | P3 | 2159.93 | ● | 0% | ● | 0% | ● | 0% | ● | 0% | ● | 0% | ● | 0% |
|  | P6 | 1588.63 | ● | 0% | ● | 0% | ● | 0% | ● | 0% | ● | 0% | ● | 0% |
|  | P9 | 1616.03 | ● | 0% | ● | 0% | ● | 0% | ● | 1.63% | ● | 0% | R | 2.44% |
|  | P12 | 4328.44 | ● | 0% | ● | 0% | ● | 0% | I | 4.53% | ● | 0% | R | 5.73% |
|  | P15 | 1759.38 | L | 7.20% | ● | 1.42% | ● | 0% | I | 11.05% | I | 5.11% | R | 29.10% |
|  | P18 | 5180.05 | ● | 0% | ● | 1.80% | ● | 2.86% | I | 58.25% | ● | 0% | R | 61.43% |
| Lineage **3** passages | P3 | 2225.56 | ● | 0% | ● | 0% | ● | 0% | ● | 0% | ● | 0% | ● | 0% |
|  | P6 | 2724.76 | ● | 0% | ● | 0% | ● | 0% | ● | 0% | ● | 0% | ● | 0% |
|  | P9 | 3654.99 | ● | 0% | ● | 0% | ● | 0% | ● | 0% | ● | 0% | R | 1.68% |
|  | P12 | 1915.65 | L | 3.02% | ● | 0% | N | 2.47% | I | 8.63% | I | 2.68% | R | 21.34% |
|  | P15 | 1505.72 | ● | 0% | ● | 0% | ● | 0% | I | 16.70% | ● | 0% | R | 24.20% |
|  | P18 | 1505.45 | ● | 0% | A | 1.65% | N | 4.27% | I | 56.04% | ● | 0% | R | 59.33% |

| <5% | 5-24% | 25-49% | 50-74% | 75-100% |
| --- | --- | --- | --- | --- |

**SUPPLEMENTARY FIGURES**


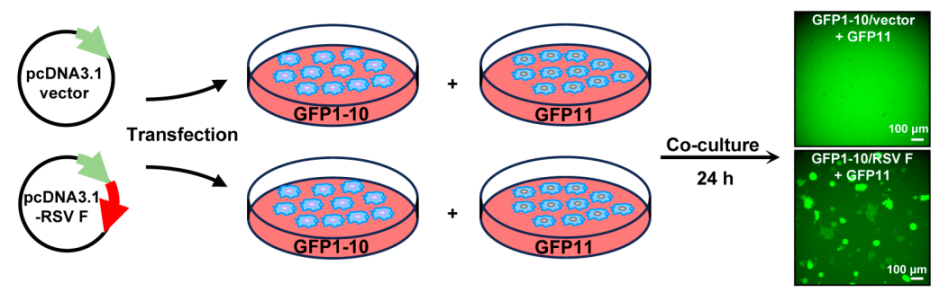


**SUPPLEMENTARY FIG 1 RSV F mediates cell‒cell fusion.** Schematic representation of the GFP-split fusion model for cell‒cell fusion mediated by the RSV F protein. HEK293T cells expressing either GFP1-10 or GFP11 (HEK293T-GFP-split cells) were transfected with RSV F or pcDNA3.1-empty control plasmids, and fusion was detected by measuring GFP fluorescence after 24 hours of co-culture. The graphs shown are representative of three independent experiments.

**
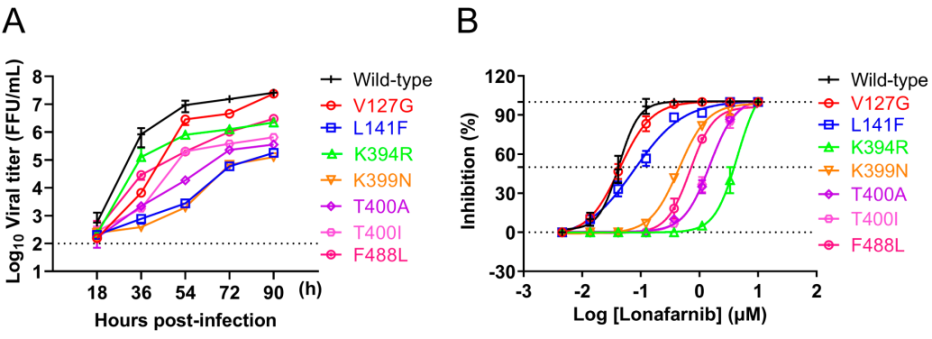
**

**SUPPLEMENTARY FIG 2 Validation of identified mutations in recombinant RSV. (A**) Growth curves of wild-type RSV and its variants with single F protein mutations. HEp-2 cells were infected individually with RSV or its variants. At the indicated time points after viral infection, the virus suspensions were harvested and subjected to viral titer determination via FFA. (**B**) Curves of the inhibition of recombinant live RSV harboring single F protein mutations by lonafarnib. Representative curves from a single experiment of three biologically independent experiments are shown. The error bars denote the means ± SDs of three technical replicates.

**
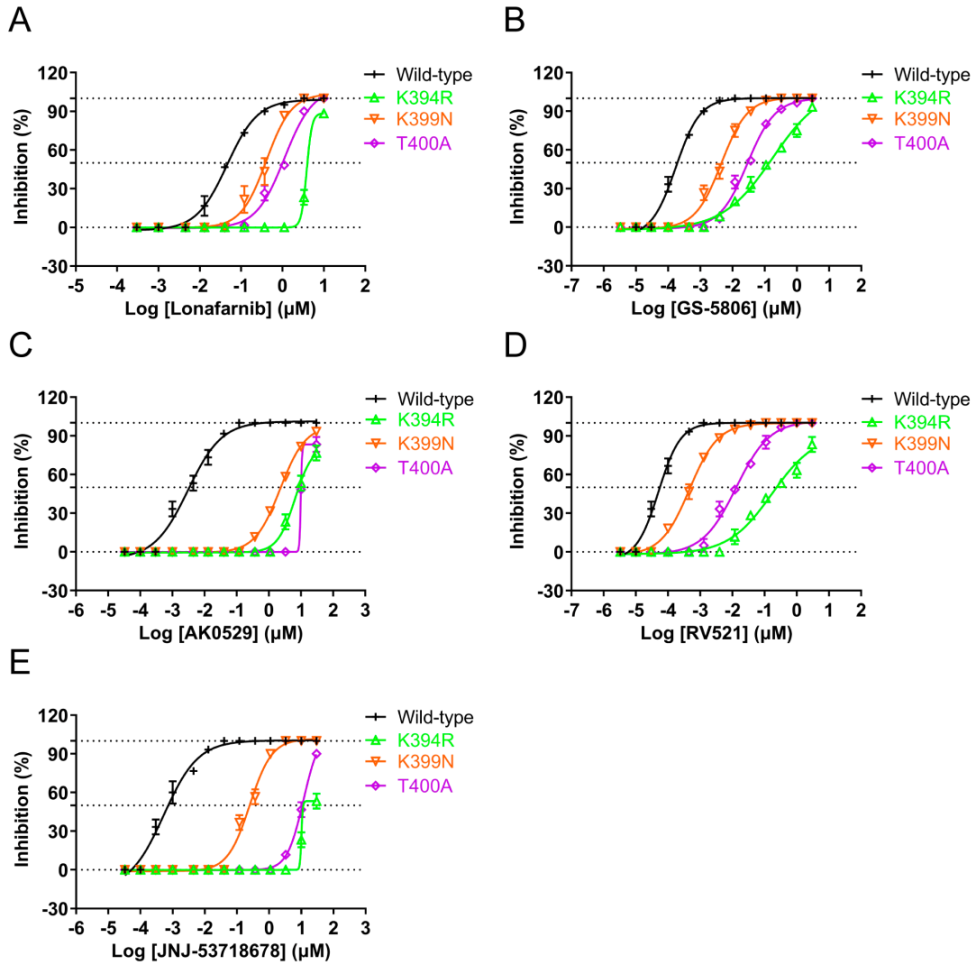
**

**SUPPLEMENTARY FIG 3 Individual inhibition curves of recombinant live RSV carrying single F protein mutations by lonafarnib (A), GS-5806 (B), AK0529 (C), RV521 (D), and JNJ-53718678 (E).** Representative curves from a single experiment of three biologically independent experiments are shown. The error bars denote the means ± SDs of three technical replicates.

**
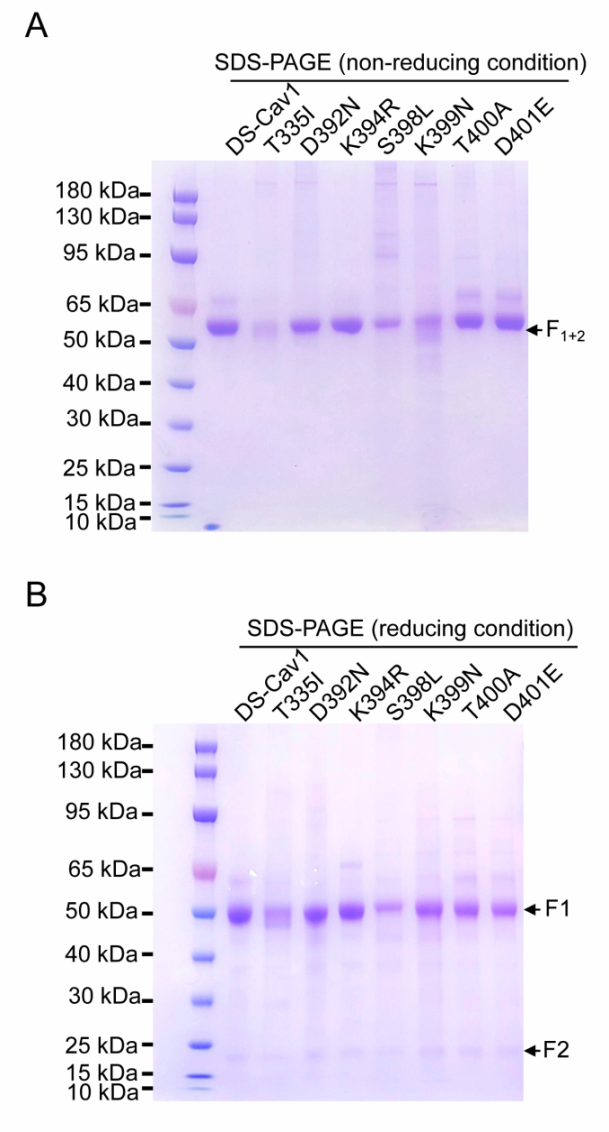
**

**SUPPLEMENTARY FIG 4 Analysis of purified prefusion-stabilized RSV F variant DS-Cav1 and its mutants by SDS–PAGE**. (**A**) Non-reducing condition. (**B**) Reducing condition. The images shown are representative of three independent experiments.

**
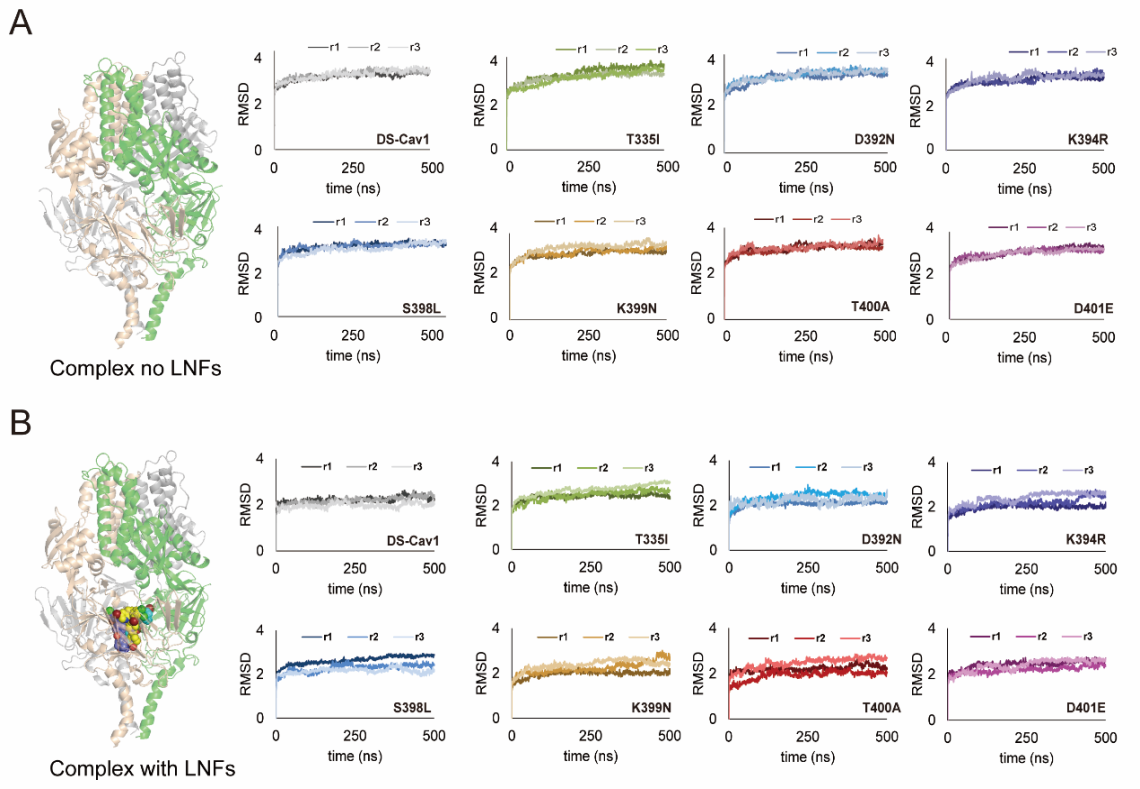
**

**SUPPLEMENTARY FIG 5** RMSD plots of the RSV-F MD simulation over time. Complex model without (**A**) or with lonafarnib (**B**).

**
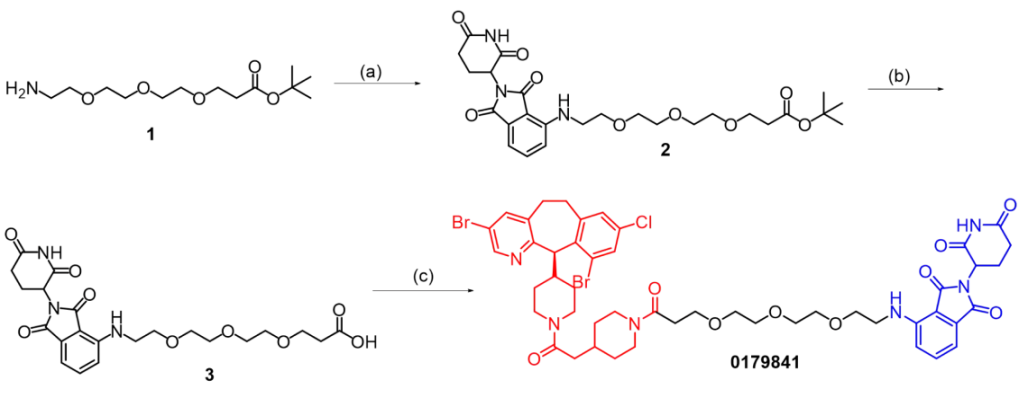
**

**SUPPLEMENTARY FIG 6 Synthesis of lonafarnib derivative-PROTAC**. Reaction conditions: (a) 2-(2,6-Dioxopiperidin-3-yl)-4-fluoroisoindoline-1,3-dione, DIPEA, DMSO, 90 ℃, 2.5 hours, 19%; (b) TFA, DCM, 0 ℃ to r.t., 2 hours, 42%; (c) (*R*)-1-(4-(3,10-dibromo-8-chloro-6,11-dihydro-5H-benzo[5,6]cyclohepta[1,2-b]pyridin-11-yl)piperidin-1-yl)-2-(piperidin-4-yl)ethan-1-one, HATU, DIPEA, DMF, 3 hours, 46%.

**
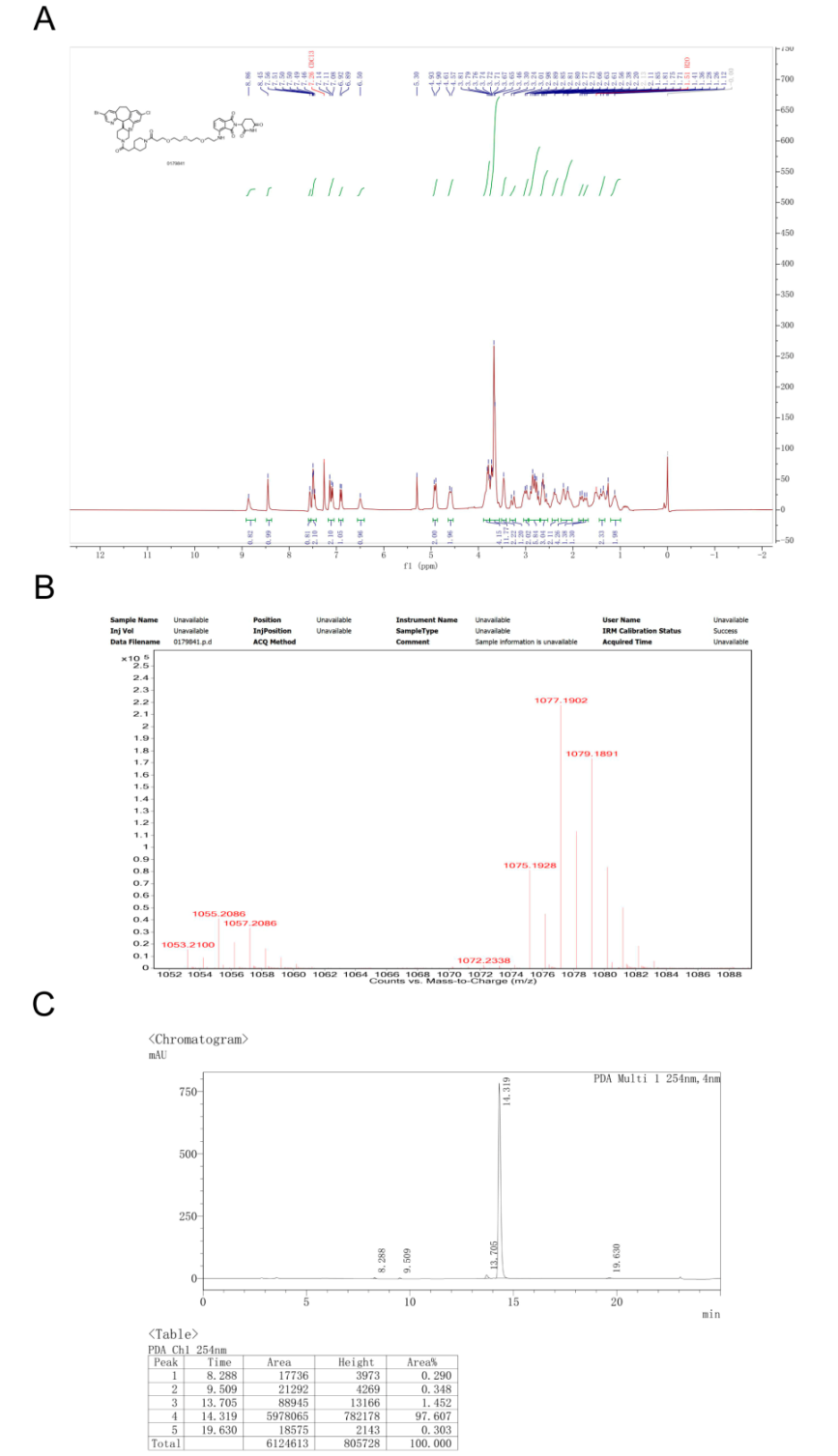
**

**SUPPLEMENTARY FIG 7 Analytical data (^1^H NMR, HRMS, and HPLC traces). (A**) **^1^H NMR** (300 MHz) spectrum of compound 0179841 in CDCl_3_. (**B**) HRMS of compound 0179841. (**C**) HPLC chromatogram of compound 0179841.


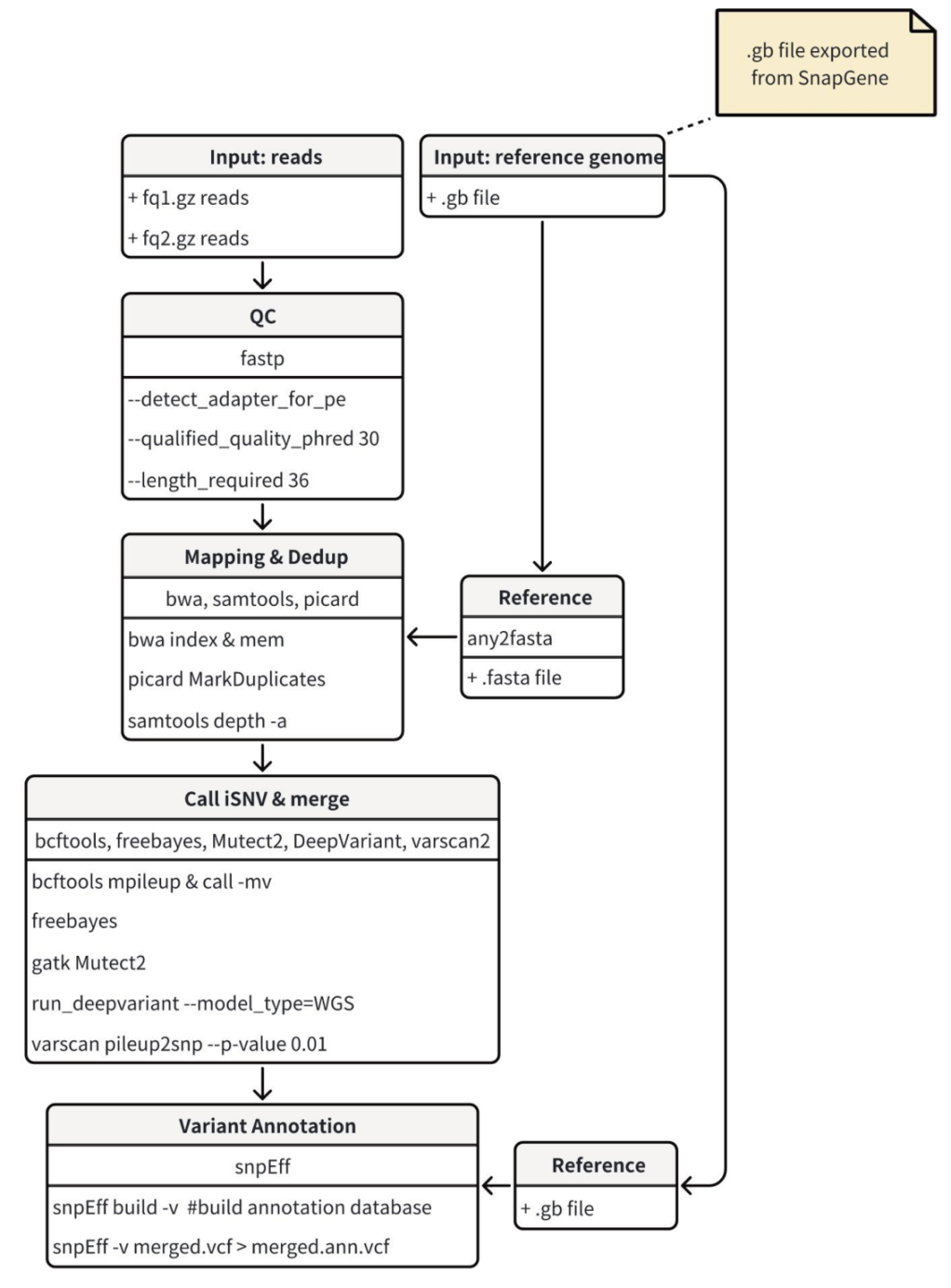


**SUPPLEMENTARY FIG 8** **Workflow diagram for RSV single nucleotide variant (SNV) calling.**
